# Phase‐Transition‐Cycle‐Induced Recrystallization of FAPbI3 Film in An Open Environment Toward Excellent Photodetectors with High Reproducibility

**DOI:** 10.1002/advs.202204386

**Published:** 2022-10-17

**Authors:** Meng Wang, Fengren Cao, Linxing Meng, Min Wang, Liang Li

**Affiliations:** ^1^ School of Physical Science and Technology Jiangsu Key Laboratory of Thin Films Center for Energy Conversion Materials & Physics (CECMP) Soochow University Suzhou 215006 P. R. China

**Keywords:** perovskites, phase transition cycle, photodetectors, recrystallization, reproducibility

## Abstract

Perovskite is an attractive building block for future optoelectronic applications. However, the strict fabrication conditions of perovskite devices impede the transformation of lab techniques into commercial applications. Here, a facile annealing‐free posttreatment is proposed to reconstruct the perovskite film to obtain high‐performance photodetectors with an optimized production rate. With posttreatment by methylamine thiocyanate, the prefabricated formamidinium‐lead triiodide (FAPbI_3_) film will undergo a recrystallization process consisting of a repeating phase‐transition‐cycle (PTC) between the black and yellow phases of FAPbI_3_, which improves the crystal quality and eliminates defects. As a result, some casually prepared or even decomposed perovskite films can be reconstructed, and the dispersion degree of the device performance based on the posttreatment method decreases by ≈21% compared to the traditional antisolvent method. This facile and annealing‐free posttreatment will be an attractive method for the future industrial production of perovskite devices.

## Graphical abstract

A novel recrystallization process is employed to optimize the fabricating process of formamidinium‐lead triiodide (FAPbI_3_) film. With a facile posttreatment by methylamine thiocyanate, FAPbI_3_ crystal will proceed a repeating phase transition cycle, which improves its crystallization and stability. Meanwhile, the film reproducibility is significantly improved by 21% in the standard deviation.

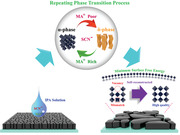

## 1 Introduction

Perovskites have been widely employed in optoelectronic fields due to their strong light absorption and high carrier mobility.^[^ ^1^, ^2^, ^3^, ^4^, ^5^, ^6^, ^7^, ^8^, ^9^, ^10^, ^11^, ^12^, ^13^, ^14^ ^]^ With the realization of high‐performance perovskite devices in the laboratory, commercial applications are also highly desired. Commercial perovskite devices demand a production line with mature techniques and a high reproduction rate to satisfy the modern industry.^[^ ^15^, ^16^ ^]^ Meanwhile, the fabrication process should be simplified with a low conditional requirement to minimize the cost.^[^ ^17^, ^18^ ^]^ However, the laboratory‐scale fabrication process often requires a carefully controlled environment, which requires a high cost to maintain ideal experimental conditions.^[^ ^19^, ^20^ ^]^ In this situation, the achievements in the laboratory are difficult to apply to societal life, and correlational research remains a hot topic.^[^ ^21^, ^22^, ^23^ ^]^

Most state‐of‐the‐art perovskite devices are fabricated by solution‐based methods, which require several steps, such as spin‐coating and annealing, to transform the precursor solution into a final perovskite film.^[^ ^24^, ^25^, ^26^, ^27^, ^28^, ^29^, ^30^ ^]^ However, these steps are very sensitive to external conditions such as temperature^[^ ^31^, ^32^ ^]^ or atmosphere.^[^ ^33^, ^34^ ^]^ Furthermore, mechanical errors such as disturbance in the time point or the volume of dropping antisolvent will also affect the final device.^[^ ^35^ ^]^ These problems bring many uncertain influence factors into the producing line, which seriously impede the development of solution‐based perovskites. Posttreatment is an attractive method to avoid the aforementioned confusions, where a recrystallization process will reconstruct the prefabricated perovskite film.^[^ ^36^, ^37^, ^38^, ^39^, ^40^ ^]^ Thus, the final perovskite quality will be determined by the recrystallization process despite the initial prefabricated perovskite film. In this situation, the complex influencing factors during the fabrication of the perovskite film can be ignored, and only posttreatment should be considered. Previous works have reported different kinds of methods to reconstruct the perovskite film; however, these methods also bring additional condition requirements, such as a special gas environment^[^ ^36^ ^]^ or high‐temperature annealing,^[^ ^39^ ^]^ which complicate the production line again. A simple and efficient secondary treatment should be found to simplify the fabrication of perovskite devices.

Here, we report a phase‐transition‐cycle (PTC)‐induced recrystallization phenomenon in a pure formamidinium‐lead triiodide (FAPbI_3_) film, which can proceed in an open environment and be controlled by adjusting the relative humidity (RH). This method can fabricate high‐quality perovskite films with a high production rate, where the fabrication block can be a casually prepared perovskite film or even a decomposed film. Methylamine thiocyanate (MASCN) is employed to treat the black phase FAPbI_3_ (*α*‐FAPbI_3_) film, and it is found that the SCN^−^ group has two reverse effects on the phase transition between the black (*α*‐) and yellow (*δ*‐) phases of FAPbI_3_, which is determined by the concentration of the MA^+^ group. Different diffusing behaviors of the two groups cause an interesting PTC between *α*‐FAPbI_3_ and *δ*‐FAPbI_3_ inside the film, which helps reconstruct the perovskite crystal with the improvement of crystallization and elimination of defects. The lateral photodetector based on the recrystallized film shows a high responsivity (*R*) of 1.44 A W^−1^ and outstanding stability. Furthermore, this PTC method can ignore the labile factors during the preceding solvent process and decrease the performance dispersion degree of the fabricated optoelectronic device by 21%. This work provides a novel approach for the potential commercial application of perovskite devices.

## 2 Results and Discussion

**Figure 1:**
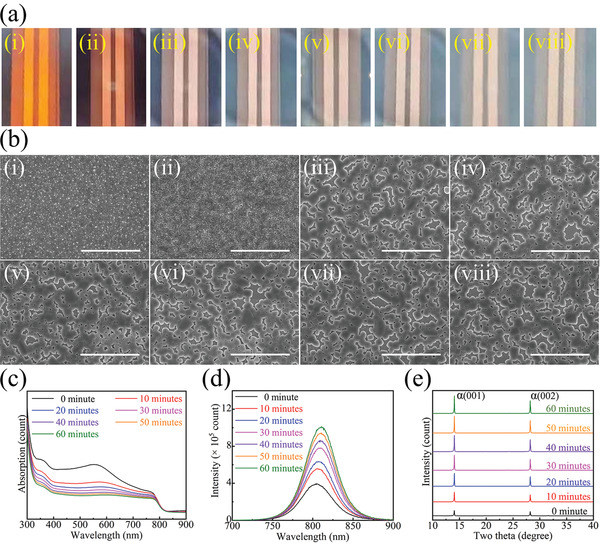
a) Back‐light and b) corresponding SEM image of the FAPbI_3_ film with different treatments, where (i) is the pristine film and from (ii) to (viii) is the MASCN‐treated film after 0, 10, 20, 30, 40, 50, and 60 minutes, respectively. The scale bar is 10 µm. c) UV–visible absorption, d) PL spectra and e) XRD results of the MASCN‐treated film after different times.

The FAPbI_3_ film is fabricated by a one‐step antisolvent method (details are provided in the Experimental Section), and the initial film without posttreatment is denoted Pristine‐FAPbI_3_. The back‐light image shows that Pristine‐FAPbI_3_ is a deep brown film (**Figure** **1**a, graph i), where the corresponding scanning electron microscopy (SEM) image (Figure 1b, graph i) indicates that the film is a compact film without holes. Then, the film is posttreated by spin‐coating with a drop of MASCN/isopropanol (IPA) solution (10 mg mL^−1^) and exposed to an air environment. Here, the film exposed for *x* minutes is denoted *x* min‐FAPbI_3_ and characterized with a time gap of 10 min from 0 to 60 minutes (corresponding to the graph from ii to viii in Figure 1a,b). The initial film (0 min‐FAPbI_3_) is still visually deep brown (Figure 1a, graph ii); however, the SEM image shows that its morphology has become mesoporous (Figure 1b, graph ii). Over time, the film becomes rough and transparent, where the backlight turns from brown to white (Figure 1a, the graph from iii to viii) and the morphology changes dramatically from a mesoporous film to large connecting crystals with holes among them (Figure 1b, the graph from iii to viii). The bright‐field optical microscope (BFOM) images exhibit a continuous in situ variation of the film, where small crystals gradually merge into a large one with holes emerging (Figure S1, Supporting Information, the graphs from i to viii correspond to the film from i to viii in Figure 1a,b). The variation spontaneously proceeds under an ambient environment, so some in situ characterizations can be employed to study this process. Ultraviolet (UV)‐visible spectroscopy was employed to observe the in situ changes in film absorption from 0 min FAPbI_3_ to 60 min FAPbI_3_ (Figure 1c). The absorption edges indicate that the bandgap of the film does not change, while the absorption intensity gradually decreases to a flat line, which is caused by the emerging pores on the film. However, the photoluminescence (PL) intensity of 60 min‐FAPbI_3_ increases to several times stronger than that of 0 min‐FAPbI_3_ (Figure 1d), indicating that carrier recombination is prevented and that the trap states are eliminated in this process (for more discussion, see Supplementary Note 1, Supporting Information). The X‐ray diffraction (XRD) patterns support this modification effect (Figure 1e). From 0 min FAPbI_3_ to 60 min FAPbI_3_, the XRD signal intensity keeps increasing, indicating the crystallization is gradually strengthened. The diffraction angle before 21° is further scanned with a tiny step of 0.002°, and the results are shown in Figure S2 (Supporting Information). The color map of intensity shows the normalized intensity of the (001) peak of *α*‐FAPbI_3_ at 14°, and its full width at half maximum (FWHM) gradually decreases, indicating a continuous modification of crystallization (Figure S2a, Supporting Information). In Figure S2b (Supporting Information), the intensity ratio between the (001) facet at 14° and the (011) facet at 20° is compared to exhibit the crystal orientation. As the intensity of (001) is normalized as 1, the value of (011) gradually decreases, indicating an increasing crystal orientation along the (001) facet.^[^ ^41^, ^42^ ^]^ The characterizations of Pristine‐FAPbI_3_ without treatment are shown in Figure S3a (PL) and S3b (XRD), Supporting Information, as a reference, and there are no variations (Supporting Information). All these results indicate that after posttreatment by MASCN, the FAPbI_3_ film experiences a recrystallization process, which changes its morphology and optimizes its crystallization.

**Figure 2:**
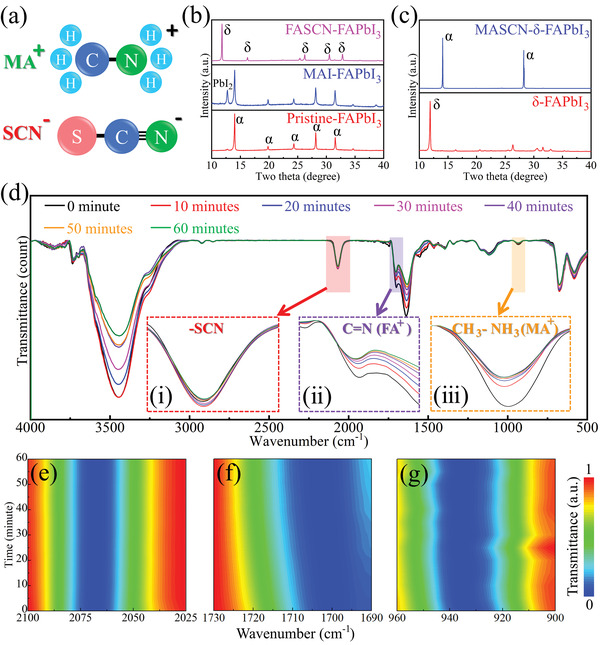
a) Molecular structure of MASCN. b) XRD patterns of Pristine‐, MAI‐treated‐ and FASCN‐treated‐FAPbI_3_ films. c) XRD patterns of the *δ*‐FAPbI_3_ film before and after MASCN treatment. d) FTIR spectra of MASCN‐treated FAPbI_3_ for different time points. The inset graph shows the details of the spectral ranges of (i) 2100–2025 cm^−1^, (ii) 1730–1690 cm^−1^, and (iii) 960–900 cm^−1^. e‐g) Color map of the spectra corresponding to inset graphs (i), (ii), and (iii).

The Pristine‐FAPbI_3_ film without treatment does not tend to vary so quickly even in a humid environment; thus, the effect of MASCN should be evaluated. The chemical components of the MASCN molecule are shown in **Figure** **2**a, which consists of an MA^+^ ion and an SCN^−^ ion. They will be evaluated individually, where methylamine iodine (MAI) and formamidine thiocyanate (FASCN) are chosen to replace MASCN to treat the FAPbI_3_ film because FA^+^ and I^−^ ions have tiny effects on FAPbI_3_. For the film posttreated by MAI, the XRD pattern shows that there is merely a difference from the initial films, and only the signal of PbI_2_ arises, which is caused by the decomposition of some produced MAPbI_3_ while annealing (Figure 2b, MAI‐FAPbI_3_). For the film posttreated by FASCN, interestingly, the film transforms from the *α*‐phase to the *δ*‐phase (Figure 2b, FASCN‐FAPbI_3_). Further experiments showed that posttreatment with formamidine iodide (FAI) did not cause a phase transition (Figure S4, Supporting Information); thus, such a phase transition was caused by the SCN^−^ group. Grätzel and his coworkers reported that MASCN can cause a phase transition of FAPbI_3_ from *δ*‐phase to *α*‐phase by surface interaction,^[^ ^43^ ^]^ and our posttreatment method on *δ*‐phase FAPbI_3_ film verifies this phenomenon (Figure 2c). However, our reference experiment on FASCN shows that SCN^−^ causes a reversed‐phase transition from the *α*‐phase to the *δ*‐phase. To evaluate the difference between MASCN and FASCN, posttreatment with different concentrations was applied. For the posttreatment with MASCN, high concentrations (20 mg mL^−1^ and 10 mg mL^−1^, Figure S5a,b, Supporting Information) optimized the crystallization of FAPbI_3_ in accordance with the results in Figure 1e. However, when the concentration is low (5 mg mL^−1^ and 2.5 mg mL^−1^, Figure S5c,d, Supporting Information), the *α*‐phase FAPbI_3_ film transforms into *δ*‐phase FAPbI_3_. The film treated with FASCN will cause the transition from the *α*‐phase to the *δ*‐phase despite the treatment concentration (Figure S6, Supporting Information). Thus, it is concluded that the SCN^−^ group will cause only an *α* to *δ* phase transition when individually, but when MA^+^ is involved, the effect will depend on the treatment concentration. As a result, the effect of MASCN can be divided into two parts: the SCN^−^ group will dominate the phase transition effect on FAPbI_3_, and the MA^+^ ion will adjust this effect. Individual SCN^−^ will just cause the *α* to *δ* transition, and when enough MA^+^ ions are involved, the effect of SCN^−^ becomes reversed, causing a *δ* to *α* transition. Phase transition is a normal and important behavior in crystal materials including perovskite, which is related to the variation of system energy.^[^ ^44^, ^45^, ^46^, ^47^, ^48^ ^]^ Previous works have reported the impacts of strain and surface interaction on the phase transition of FAPbI_3_.^[^ ^43^ ^]^ In this paper, the phase transition should be caused by the variation of lattice strain, which is affected by the interaction between FAPbI_3_ crystal and SCN^−^ group. Further discussion of the different phase transition behaviors is presented in Supplementary Note 2 (Supporting Information).

In situ time‐dependent Fourier transform‐transform infrared spectroscopy (FTIR) was employed to further characterize the behavior of MASCN (Figure 2d). Three wavenumber ranges were selected to represent the three organic groups during the variation process. They are wavenumbers of 2100–2025 cm^−1^ (stretch of C≡N, SCN^−^ group, inset graph i), 1730–1690 cm^−1^ (stretch of C=N, FA^+^ group, inset graph ii), and 960–900 cm^−1^ (twist of CH_3_‐NH_3_^+^, MA^+^ group, inset graph iii).^[^ ^49^ ^]^ The normalized variations are depicted in the color map in Figure 2e‐g. In Figure 2e, the signal of SCN^−^ has no change with time, neither the peak position nor FWHM. This result means that the chemical environment of SCN^−^ did not change during this process. Considering that MASCN is spin‐coated on the surface of the perovskite film, the SCN^−^ group keeps its initial position, where it stays at the surface of the perovskite crystal. In Figure 2f, a continuous redshift is observed for the C=N bond, indicating that the chemical environment of FA^+^ group has a continuous variation. In Figure 2g, the signal of the MA^+^ group has an obvious redshift for the first 10 min and then remains in a tiny disturbance, indicating that it is not static. The behaviors of MA^+^ and FA^+^ can be understood as follows: during the recrystallization process, the MA^+^ ions will quickly penetrate into the crystal of the FAPbI_3_ film and then diffuse, resulting in the disturbance in Figure 2g. This diffusion also causes a continuous doping process for the crystal, which can be confirmed by the continuous peak shift toward a large degree in the XRD patterns (Figure S7, Supporting Information). Previous studies have reported that the doping of SCN^−^ will not cause a degree shift,^[^ ^50^, ^51^ ^]^ so the shift must be caused by the doping of MA^+^ ions. The MA^+^ ion diffuses deeper, thus causing continuous doping of the FAPbI_3_ film and resulting in a continuous peak shift for the FA^+^ group, as shown in Figure 2f. In conclusion, the in situ time‐dependent FTIR spectra show that the SCN^−^ group will stay at the surface of the film, while MA^+^ will continue to diffuse in the crystal. Further treatment is executed on the MASCN‐treated film to observe the different behaviors of the MA^+^ and SCN^−^ groups, where the film is washed with pure IPA. This process can wash the MA^+^ and SCN^−^ ions on the surface while the MA^+^ ions inside the film remain, and the FTIR spectra in Figure S8 (Supporting Information) show that the intensity of SCN^−^ and MA^+^ ions obviously decrease after IPA washing. Here, etched X‐ray photoelectron spectroscopy (XPS) characterization at different depths is employed to verify the diffusion process. For the 0 min‐FAPbI_3_ film after IPA washing, compared to the surface of the film, there is a large shift of 1.1 eV toward a high binding energy for the film etched with 10 nm for both Pb (Figure S9a, Supporting Information) and I elements (Figure S9b, Supporting Information), Supporting Information. This is caused by a large MA^+^ content near the surface, where the MA^+^ group does not diffuse into the deep film because the recrystallization process has not begun (Figure S10a, Supporting Information). The etched depths of 20, 30, and 40 nm showed no differences, proving that the MA^+^ group was not reached here. However, for the 10 min‐FAPbI_3_ film after IPA washing, MA^+^ ions diffused into deeper crystals (Figure S10b, Supporting Information). The shift (0.2 eV) is small at 10 nm depth (Figure S11a of Pb and S11b of I, Supporting Information); meanwhile, there is also a tiny shift from a depth of 10 nm to 20 nm of 0.1 eV, exhibiting graded MA^+^ doping. The SCN^−^/MA^+^ intensity ratio was obviously larger in 0 min FAPbI_3_ than in 10 min FAPbI_3_ after IPA washing (Figure S12, Supporting Information). These results further prove that there is diffusion of MA^+^ during the recrystallization process.

**Figure 3:**
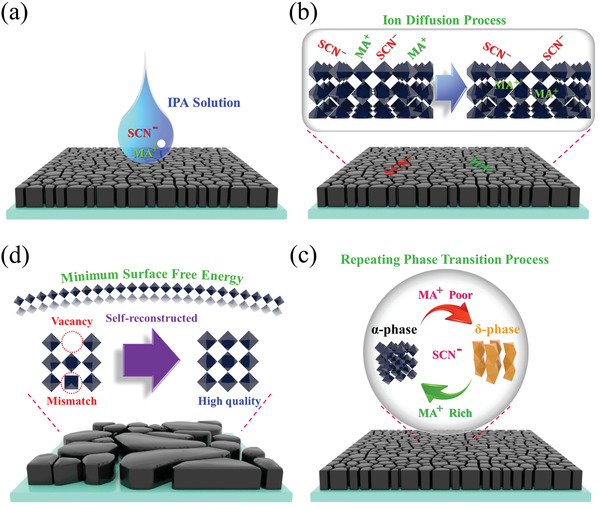
a) Coating of MASCN solution, b) ion diffusion process, c) PTC process and d) the resulting FAPbI_3_ film.

Combining the analysis of the different effects and behaviors of the MA^+^ and SCN^−^ groups, the behaviors of MASCN are depicted in Figure S13 (Supporting Information). Initially, the MA^+^ and SCN^−^ groups are spin‐coated on the surface of the FAPbI_3_ film (Figure S13a. Supporting Information). Then, the MA^+^ group diffuses into the film, while the SCN^−^ group stays at the surface (Figure S13b, Supporting Information). During the diffusion process, there will be a dynamic distribution of MA^+^ groups. In the MA‐rich region, the SCN^−^ group will cause a transition from *δ*‐phase to *α*‐phase; in the MA‐poor region, the SCN^−^ group will cause a transition from *α*‐phase to *δ*‐phase. With the continuous diffusion process of the MA^+^ group, there will be microscale phase transition cycles, which reconstruct the FAPbI_3_ film (Figure S13c, Supporting Information). The XRD patterns prove such a coexisting situation for these two phases when the film is recrystallized with MASCN at high concentrations (Figure S14, Supporting Information). The XRD patterns from 10 to 15° show that the signal of *δ*‐phase (11.8°) also gradually emerges with the strength of *α*‐phase (14°). Based on the aforementioned discussion, the whole recrystallization process is depicted in **Figure** **3**. Initially, the MASCN/IPA solution is spin‐coated on the surface of the FAPbI_3_ film (Figure 3a). With the evaporation of IPA, the MA^+^ and SCN^−^ ions will adsorb on the crystal surface, and then MA^+^ ions will diffuse into the crystal while SCN^−^ will stay at the surface (Figure 3b). The imbalanced distribution of MA^+^ will cause a reverse effect of SCN^−^ at the microscale, which results in a repeating PTC process (Figure 3c). The repeating PTC will result in a slow recrystallization process, where the crystal will be reconstructed. The crystal system is intended to minimize the surface free energy to reach a stable construction. Compared to the cubic structure, the spherical crystal has a smaller surface/volume ratio, which is favorable for minimizing the system energy. Thus, the film will convert from a flat film with cubic crystals to a mesoporous film with continuous spherical crystals (Figure 3d). The slow recrystallization also helps reduce the vacancies and crystal mismatches, which results in better crystallization.

**Figure 4:**
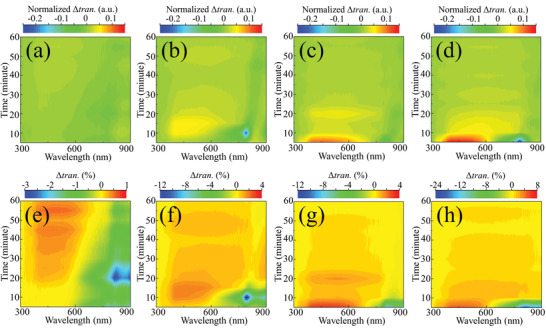
a‐d) The normalized and e‐h) absolute values of Δtran. under different RHs, which are a,e) 0%–5%, b,f) 20%–25%, c,g) 50%–55% and d,h) 80%–85%, respectively.

Moisture in the air will stimulate the *α* to *δ* transition, which may be employed to control the speed of the recrystallization process. In the PTC process, the moisture in background will facilitate the phase transition from *α*‐ to *δ*‐ phase. On the contrary, the increasing amount of *δ*‐phase will also accelerate the phase transition from *δ*‐ to *α*‐ phase. As a result, the speed of PTC process is accelerated (Figure S15, Supporting Information). The transmittance spectrum is an ideal signal to monitor the recrystallization process considering the obvious morphology change. Here, Δtran. is defined as the transmittance variation of the film between two time points. For example, Δtran._10_ = transmittance_10_ – transmittance_0_, which presents the variation of transmittance from 0 min FAPbI_3_ to 10 min‐FAPbI_3_. The variation of the MASCN‐treated FAPbI_3_ film under a sealed environment is first observed to block its contact to external stimulation, where the film is dipped into chlorobenzene (CB), which is very stable with perovskite. The variation still occurs, which indicates that this process is spontaneous even though very slow (Figure S16a,b, Supporting Information). However, the film variation under an open environment is quick and fierce (Figure S16c,d, Supporting Information). Therefore, the recrystallization process can proceed spontaneously, and moisture in air can exactly accelerate the process. The variation in transmittance under different RHs (0%–5%, 20%–25%, 50%–55%, and 80%–85%) is shown in Figure S17 (Supporting Information), and the calculated Δtran. is shown in **Figure** **4**. From Figure 4a‐d, Δtran. is calculated with the normalized value of transmittance to compare their differences under different RHs. When the film is under a low RH of 0%–5%, the variation is small and slow, similar to the film sealed in CB. However, when RH increases, the variation becomes large and harsh. Under RH values of 80%–85%, a sharp increase in Δtran. occurs within 10 min. The absolute value of Δtran. is also depicted in Figure 4e‐h, which corresponds to the film from Figure 4a‐d. With an exposure time of 60 min, transmittance increases by <1% for RH values of 0%–5% and by ≈8% for RH values of 80%–85%. In this situation, the variation of the MASCN‐treated FAPbI_3_ film can be concluded: the MASCN‐treated FAPbI_3_ film will recrystallize spontaneously, but the moisture will accelerate this process, where higher RH causes quicker variation (More discussions in Note S3, Supporting Information).

**Figure 5:**
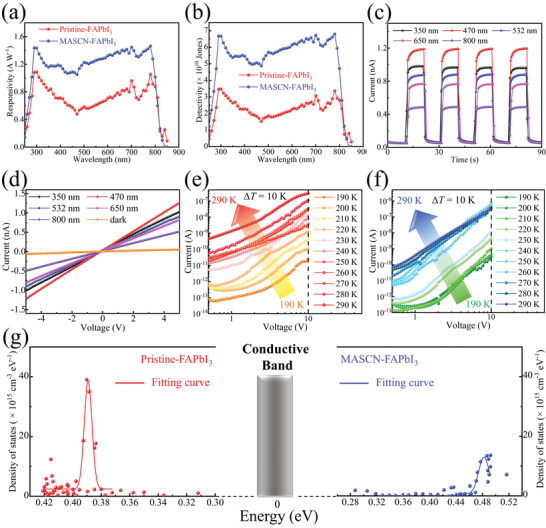
a) *R* and b) D* of the photodetector based on Pristine‐FAPbI_3_ and MASCN‐FAPbI_3_ with 1 V external bias, respectively. c) *I*–*t* and d) *I*–*V* curves of the MASCN‐FAPbI_3_‐based photodetectors. e,f) The temperature‐dependent SCLC curves for Pristine‐FAPbI_3_ and MASCN‐FAPbI_3_ films, respectively, and g) the extracted DOS distribution.

The recrystallization process in the air can be employed to improve the optoelectronic properties of the film, and the results in Figure 4 prove that this process can be adjusted by optimizing the exposure time and RH. The relative values of Δtran. at the wavelength of 500 nm under different RHs are depicted in Table S1, Supporting Information. Under a high RH over 50%, there is a drastic variation in the first ten minutes, which is not facile for controlling the PTC process. Thus, a low RH is more favorable for fabricating devices. Considering that the PTC process hardly proceeds under extremely low RH (0%–5%), we employ a RH of 20%–25% to fabricate photodetectors. A lateral photodetector is fabricated based on the recrystallization process (Figure S18, Supporting Information). In this process, IPA washing is employed to terminate the recrystallization process by eliminating the surface SCN^−^ group, which avoids additional annealing treatment (Figure S19, Supporting Information). The prepared FAPbI_3_ film (details in the Experimental Section) was treated with 10 mg mL^−1^ MASCN and then exposed to air (RH∼25%) for different times. IPA washing is then employed to stabilize the film, and a pair of Au electrodes is evaporated on the side of the film. The device based on the film with an *x*‐minute exposure time is denoted as *D_x_*_min_, and the corresponding *I*–*t* curve is depicted in Figure S20 (Supporting Information). Under illuminating, the response current increases obviously from *D*_0 min_ to *D*_10 min_ and then gradually decreases by a small magnitude (more discussion is provided in Note S4, Supporting Information). Therefore, the film treated for 10 min is chosen as the best parameter, and hereafter, the corresponding film is denoted MASCN‐FAPbI_3_. Responsivity (*R*) is widely employed to describe the ability of a photodetector to detect incident light, and it is defined as *R* = $\frac{I_{\mathrm{light}}-I_{\mathrm{dark}}}{PS}$, where *I*_light_ is the light current, *I*_dark_ is the dark current, *S* is the active area, and *P* is the intensity of incident light. The comparison between the device based on Pristine‐FAPbI_3_ and MASCN‐FAPbI_3_ is depicted in **Figure** **5**a. At an incident light of 700 nm (0.46 µW cm^−2^) and a low bias of 1 V, the MASCN‐FAPbI_3‐_based device shows an *R* of 1.44 A W^−1^, which is obviously improved compared to the device based on Pristine‐FAPbI_3_ (0.96 A W^−1^). The *I*–*V* curve in the dark is shown in Figure S21 (Supporting Information). Under dark conditions, the current is mainly attributed to the free carriers caused by vacancies and trap states, which will cause a disturbance for detecting incident light signals. Detectivity (D*) is applied to describe the ability to detect incident light signals against the noise of photodetectors, and it is defined as D* = $\frac{\sqrt{S}}{NEP}$, NEP = $\frac{I_{n}}{R\sqrt{B}}$, where In is the noise current characterized under 1 V at different frequencies (Figure S22, Supporting Information), B is the electrical bandwidth of 2 Hz,^[^ ^52^ ^]^ and the results are shown in Figure 5b. The D* of the device based on MASCN‐FAPbI_3_ can reach 1.26 × 10^11^ Jones, which is much larger than the device based on Pristine‐FAPbI_3_ of only 7.06 × 10^10^ Jones. For the optimized device based on MASCN‐FAPbI_3_, the *I–t* and *I–V* curves under different wavelengths of incident light are shown in Figure 5c,d, respectively. The device shows a good periodic response from the ultraviolet to near‐infrared region. Rise and decay time is usually employed to descript the response speed of photodetectors, which is defined as the time for photocurrent rising from 10% to 90% (rising tine) or decaying from 90% to 10% (decay time) of the peak value.^[^ ^53^ ^]^ The device has a fast rise and decay time of 73 and 74 ms, respectively (Figure S23, Supporting Information). It is also important to understand the intrinsic modification mechanism of the recrystallization process on FAPbI_3_ film. A temperature‐dependent space charge limited current (SCLC) characterization is carried out to study the variation in the energy band. Temperature‐dependent SCLC is an efficient way to extract the density of state (DOS) distribution in semiconductors, including perovskites, and the details are discussed in the Experimental Section and Note S5 (Supporting Information). The temperature‐dependent *I–V* curves are shown in Figure 5e‐f for the Pristine‐FAPbI_3_ and MASCN‐FAPbI_3_ ‐based devices, respectively, and the extracted DOS distribution is depicted in Figure 5g. The Pristine‐FAPbI_3_ film exhibits a density peak at ≈0.39 eV below the conductive band, which could be caused by PbI defects according to previous theoretical calculations.^[^ ^54^ ^]^ The density peak of MASCN‐FAPbI_3_ is at ≈0.48 eV, with a trap density less than half that of the Pristine‐FAPbI_3_ film, indicating that the recrystallization process helped heal defects. Trap states provide recombination centers for photo‐generated carriers, which will harm the response current. Under light illumination, photo‐generated carriers will be transformed into response current more efficiently in the MASCN‐FAPbI_3_ ‐based device for the fewer carrier recombination, thus its corresponding responsivity is obviously improved compared to the Pristine‐FAPbI_3_ ‐based device (Figure 5a). The stability of the film is also investigated. It is widely known that MA additives accelerate the decomposition of FA‐based perovskite films because of the hydrophilicity of the MA group.^[^ ^55^ ^]^ Although SCN− is reported to be able to stabilize perovskite crystals, our IPA washing process wiped most of them on the film surface. Interestingly, when the films with different treatments are stored in an environment with a high RH over 90%, the MASCN‐treated film still exhibits much better stability than the Pristine‐FAPbI_3_ film (Figure S24, Supporting Information). However, the MASCN‐treated film without air exposure (0 min FAPbI_3_) decomposes first, which is even faster than the Pristine‐FAPbI3 film. The difference in stability is attributed to the difference in system energy. When the film is exposed to air for some minutes, its morphology turns from a flat film to rounded large crystals (Figure 1b). The rounded shape endures the film with a small surface free energy, which is more stable than the flat film (Figure 3d). The film without air exposure still has a flat morphology; furthermore, the residue MA^+^ inside the film causes a faster decomposition than the Pristine‐FAPbI3 film. The stability of the photodetectors is investigated in Figure S25 (Supporting Information). The photodetector based on the MASCN‐FAPbI_3_ film can maintain over 80% of its initial response after storage under an RH of 50% for 30 days. As a reference, the photodetector based on Pristine‐FAPbI3 falls to no response for only 3 days.

**Figure 6:**
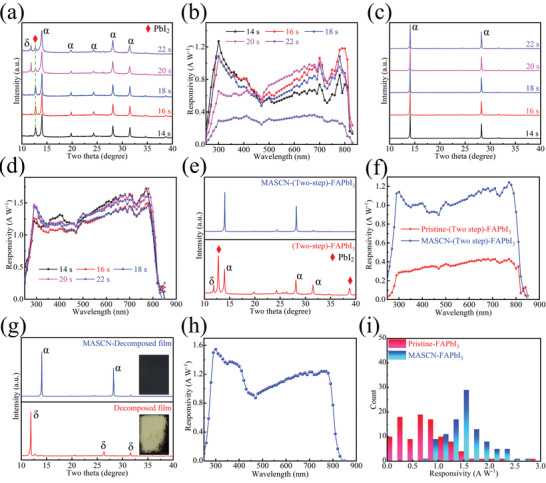
a) XRD patterns of the (*x* s)‐FAPbI_3_ film and b) *R* of the photodetector based on the corresponding film. c) XRD patterns of the MASCN‐treated (*x* s)‐FAPbI_3_ film and d) the *R* of the photodetector based on the corresponding film. e) XRD patterns of the two‐step fabricated and MASCN‐treated FAPbI_3_ film and f) the *R* of the photodetector based on the corresponding film. g) The XRD patterns of the decomposed and MASCN‐treated FAPbI_3_ film and h) the *R* of the photodetector based on the treated film. i) A statistic of the *R* of the photodetectors based on Pristine‐ and MASCN‐FAPbI_3_ with 100 devices each. All electrical measurements are measured under 1 V external bias.

There exist some tiny process windows in the operations of the solution‐based perovskite fabrication method, and the disturbances in these operations would harm the uniformity of the final device. For example, the changes at the time point of dropping antisolvent will affect the final film quality.^[^ ^35^ ^]^ Here, our PTC process can correct these disturbances, and some experiments are simulated to verify it. First, the parameters during the one‐step antisolvent method are regulated to simulate mechanical errors. The time point of dropping antisolvent is set at 14, 16, 18, 20, and 22 seconds before the end (the resulting films are denoted as (x s)‐film, where x is 14, 16, 18, 20, or 22). The XRD patterns of these films are shown in **Figure** **6**a. The (14 s)‐, (16 s)‐, and (18 s)‐films show a similar peak signal, which indicates that they are all inside the process window. However, the photodetectors based on them still perform quite differently (Figure 6b). Furthermore, the (20 s)‐films and (22 s)‐film show obvious signals of the *δ* phase, which makes their photodetectors much poorer than others. Then, each film is treated by the PTC process, and the XRD patterns of the treated films are shown in Figure 6c. The crystal qualities are all modified and uniform as high‐orientation FAPbI3 films. The morphologies of the film before and after treatment are shown in Figure S26, Supporting Information. Their initial morphologies are quite different (Figure S26a–e, Supporting Information), where different films have different crystal sizes. The modified films become uniform (Figure S26f–j, Supporting Information), and as a result, the photodetectors based on the modified films show negligible differences (Figure 6d). The result shows that our recrystallization process can correct mechanical mistakes during the fabrication of initial films. Then, the PTC method is further applied to examine its effect on different fabrication methods. In addition to the one‐step method, the two‐step method is also widely employed for fabricating FA‐based perovskite films. Here, FAPbI_3_ films are prepared by a two‐step fabrication method (details in the experimental section). The initial film shows poor crystallization with many signals of the *δ* phase and PbI2, while the PTC process also modified it successfully (Figure 6e). The morphology variation is shown in Figure S27, Supporting Information, and the corresponding photodetector shows a huge improvement after modification (Figure 6f). Finally, the PTC method is employed to save a decomposed film (Figure 6g). Normally, when the perovskite film decomposes, it is difficult to heal the decomposition, and the materials become rubbish. For example, when *α*‐FAPbI3 decomposes into *δ*‐FAPbI3, heating at 150 °C is not able to transform it back to a useful black phase. However, the PTC method successfully transforms a decomposed film into a high‐quality FAPbI3 film (Figure 6g and Figure S28, Supporting Information), and its performance is competitive with that of a normal PTC‐treated device (Figure 6h). The abovementioned results show that our PTC process can produce uniform photodetectors while ignoring the initial building blocks. A performance statistic of the Pristine‐ and MASCN‐FAPbI3‐based device is shown in Figure 6i (for *R*) with 100 devices each. Here, the standard deviation (SD) is employed to describe the dispersion degree of the devices fabricated by different methods. For the *R* in Figure 6i, the SD of the traditional method is 0.5035, and our PTC method reduces its value to 0.3969 with a decreasing percentage of 21%, which promises an attractive potential for further application.

## 3 Conclusions

In conclusion, a novel recrystallization process is introduced to obtain high‐quality FAPbI3 films with an ideal production rate. With the posttreatment of MASCN, the prefabricated FAPbI3 film undergoes a repeating phase transition cycle, which reconstructs the perovskite crystal despite the initial building block. The lateral photodetector shows a high R of 1.44 A W^−1^ under a low external bias of 1 V. Additionally, the recrystallization process can proceed in an open environment and be controlled by adjusting the RH, and the producing rate is obviously raised with a lower standard deviation of the device performance. This work will provide an attractive idea for industrial production for perovskite applications.

## 4 Experimental Section

### Materials

All materials were purchased and used without further purification. Methylamine thiocyanate (MASCN) and formamidine thiocyanate (FASCN) were purchased from Shanghai Macklin Biochemical Co. Lead iodide (PbI_2_) and [6,6]‐phenylC61‐butyric acid methyl ester (PCBM) were purchased from Xi'an Polymer Light Technology Corp. Chlorobenzene (CB), dimethylformamide (DMF), and isopropanol (IPA) were purchased from Sigma–Aldrich. N‐Methylpyrrolidone (NMP) was purchased from Aladdin Biochemical Technology Co. Formamidine iodide (FAI) was purchased from the Great Cell Solar Co. Ltd.

### Fabrication of FAPbI_3_ Films

FAI and PbI_2_ (molar ratio of 1:1) were dissolved in DMF/NMP (6:2) solution to prepare a precursor solution (1.25 M). The solution was stirred at 343 K for 10 min before use. The solution was spin‐coated onto the substrate by using a one‐step antisolvent method. In detail, 40 µL of precursor solution was coated onto the substrate and spun at 5000 rpm for 30 s. At 18 s before the end of the spinning process, 100 µL of CB was quickly spin‐coated onto the substrate. Then, the film was annealed at 424 K for 10 min to fabricate the perovskite film. All steps were carried out under N_2_ conditions.

### Posttreatment by MASCN

The MASCN solution was prepared by dissolving MASCN powder into IPA solution at room temperature. Then, the MASCN solution was spin‐coated on the prepared FAPbI_3_ film at 3000 rpm for 30 s. The treated film was further exposed in an open environment for different times. After the exposure process, the film is washed by spin coating IPA or dipping into IPA solution.

### Characterization

Film morphology images were tested by field‐emission scanning electron microscopy (SU8100, Hitachi). The matter phase was recorded by an X‐ray diffractometer (D/MAX‐III‐B‐40KV, Cu K_*α*_ radiation, *λ* = 0.15 418 nm). Fourier transform infrared (FTIR) spectroscopy was carried out using a Fourier infrared spectrometer (NICOLET 6700, BRUKER). The light absorption curve was measured by using a UV–visible spectrophotometer (Thermo Scientific, Escalab 250Xi). XPS was carried out using an ESCALAB 250Xi. The photoelectric properties of the samples were measured using a Keithley 4200 Source Meter with a monochromator (Zolix, Omni‐*λ* 3009) to generate monochromatic light. The light intensity was calibrated by using a standard Si cell. The bright‐field optical microscope (BFOM) image was recorded by an optical microscope (PSM 1000, Motic). The photoluminescence (PL) was measured using an Edinburgh instrument (LifeSpec II).

### Temperature‐Dependent SCLC Characterization

A lateral device for the SCLC characterization was fabricated by fixing an ultrathin shadow mask on the perovskite film tightly. Then, PCBM/CB solution (10 mg mL^−1^) was spin‐coated on the film at 3000 rpm for 30 s, followed by 10 min of annealing at 70 °C. Then, the film is evaporated with a 40‐nm Ag electrode. For the temperature‐dependent measurement, the device was characterized on a cryogenic probe station (Lakeshore). The temperature was controlled from 190 K to 290 K with a step of 10 K. At each temperature point, an *I*–*V* curve was measured by the Keithley 4200 Source Meter.

## Conflict of Interest

The authors declare no conflict of interest.

## Supporting information

Supporting Information

## Data Availability

The data that support the findings of this study are available from the corresponding author upon reasonable request.
