# Supplementary material for: Phase‐Transition‐Cycle‐Induced Recrystallization of FAPbI3 Film in An Open Environment Toward Excellent Photodetectors with High Reproducibility
Source: Adv Sci (Weinh). 2022 Oct 17;9(34):2204386. doi: 10.1002/advs.202204386 (PMC9731687; doi:10.1002/advs.202204386)
Supplement: Supplementary file 1 — Supporting Information [file ADVS-9-2204386-s001.pdf]

Supporting Information

Phase-transition-cycle-induced Recrystallization of $FAPbI_3$ Film in An Open Environment Toward Excellent Photodetectors with High Reproducibility

*Meng Wang, Fengren Cao, Linxing Meng, Min Wang, Liang Li**

School of Physical Science and Technology, Jiangsu Key Laboratory of Thin Films, Center for Energy Conversion Materials & Physics (CECMP), Soochow University, Suzhou 215006, P. R. China
Email: lli@suda.edu.cn

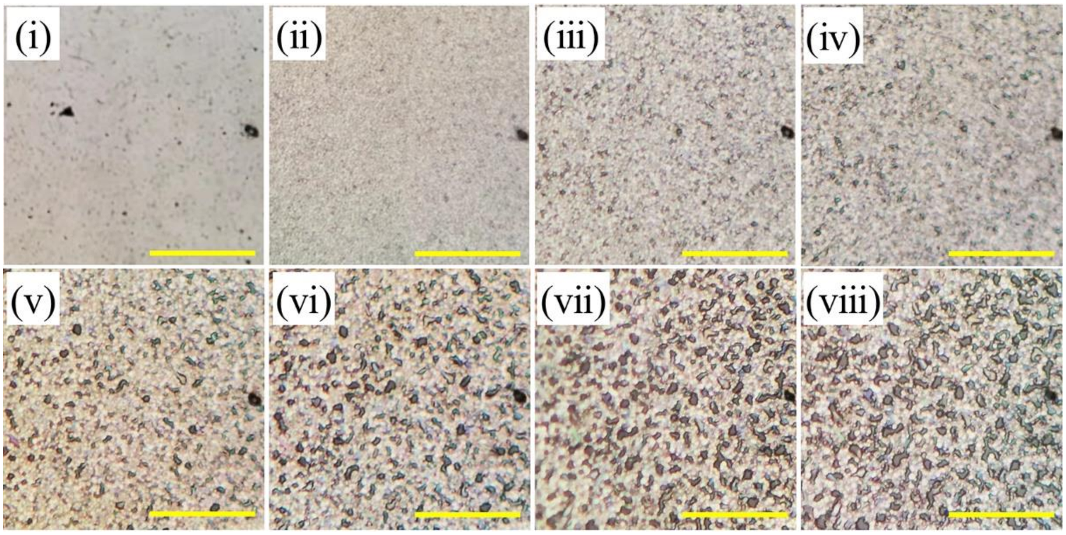

Figure S1. The BFOM images of the (ⅰ) Pristine-$FAPbI_3$ film and the *x*-min $FAPbI_3$ film from (ⅱ) 0 minutes to (ⅷ) 60 minutes. The scale bar is 50 μm.

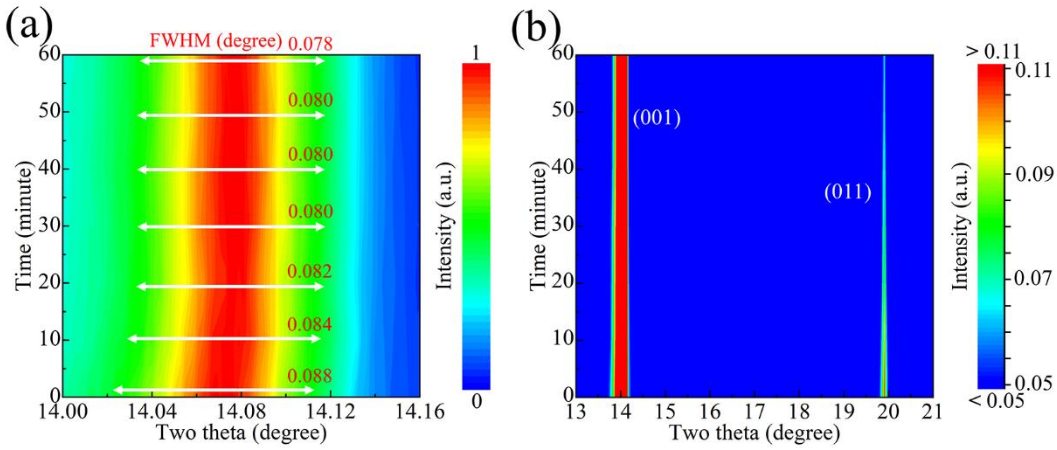

Figure S2. Color map of the XRD intensity of the *x*-min $FAPbI_3$ film. a) The details at 14° and corresponding FWHM. b) The intensity ratio between the peaks at 14° and 20°.

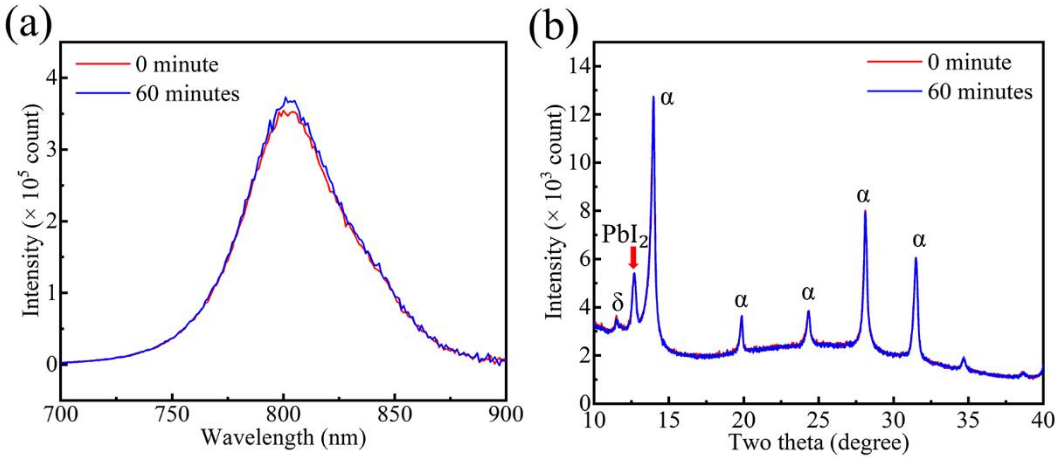

Figure S3. a) PL and b) XRD pattern of the Pristine-$FAPbI_3$ film at 0 minute and 60 minutes.

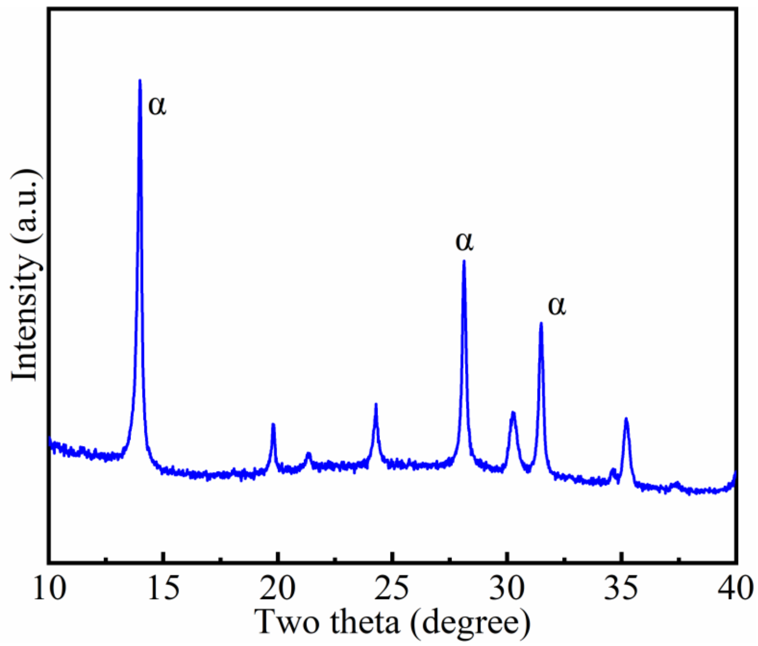

Figure S4. XRD pattern of the $FAPbI_3$ film treated by FAI of 10 mg mL^{-1} in IPA.

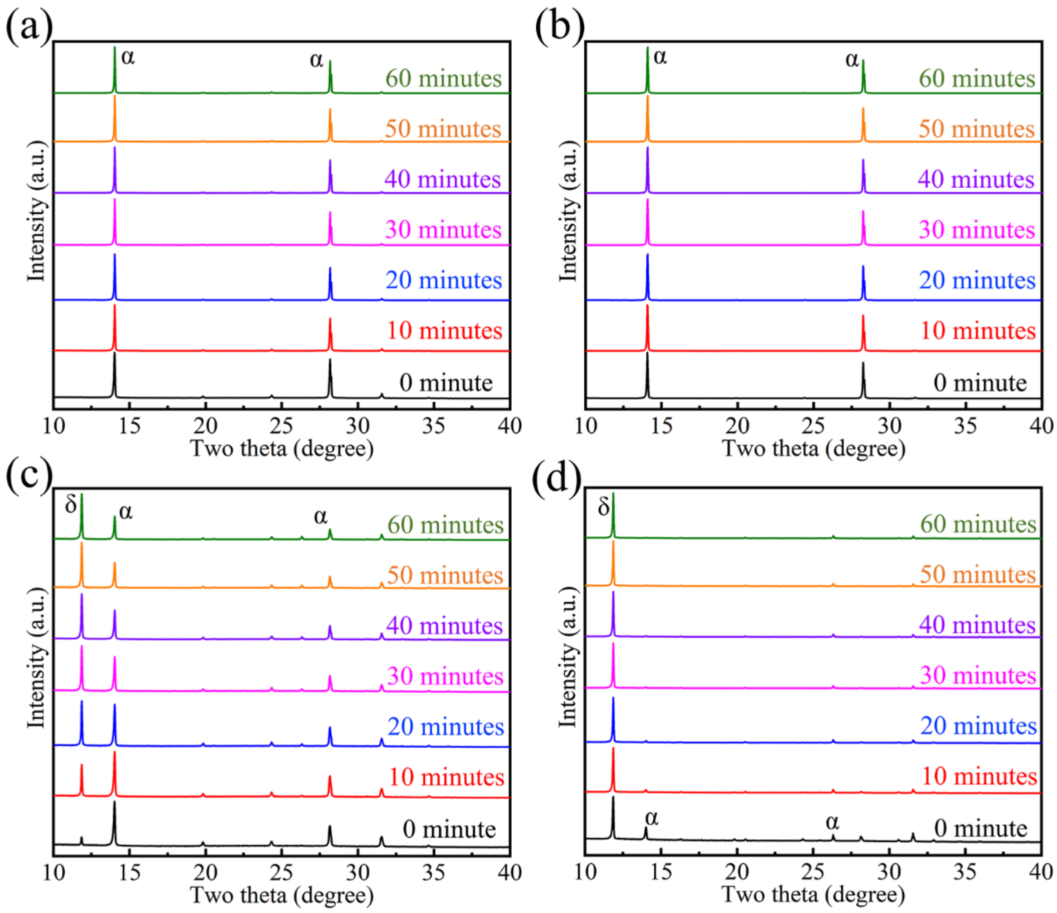

Figure S5. The time-dependent XRD characterizations on the $FAPbI_3$ film treated with MASCN of a) 20, b) 10, c) 5 and d) 2.5 mg mL^{-1}, respectively.

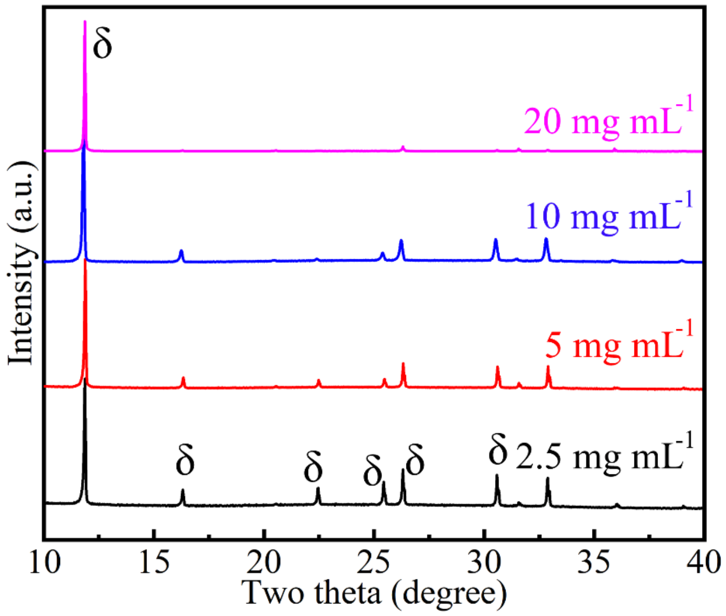

Figure S6. XRD pattern of the $FAPbI_3$ film treated with different concentrations of FASCN.

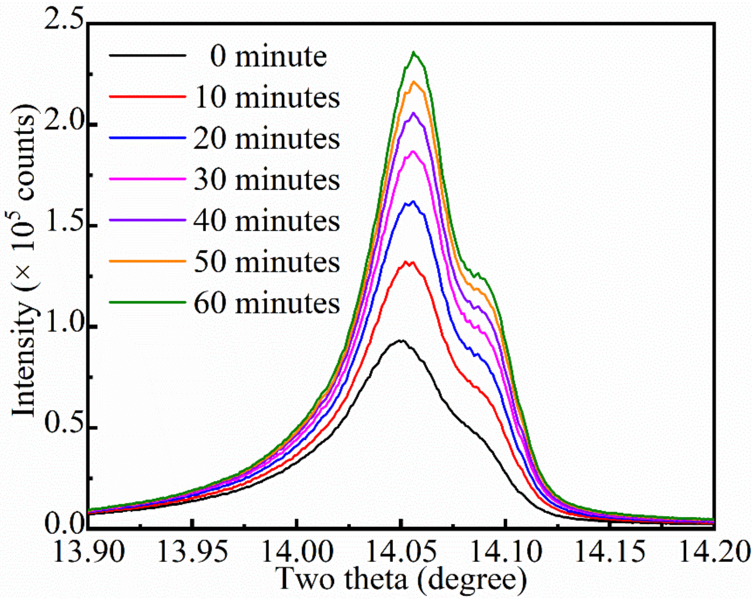

Figure S7. The narrow-scanning XRD pattern of the MASCN-treated film from 13.9° to 14.2°.

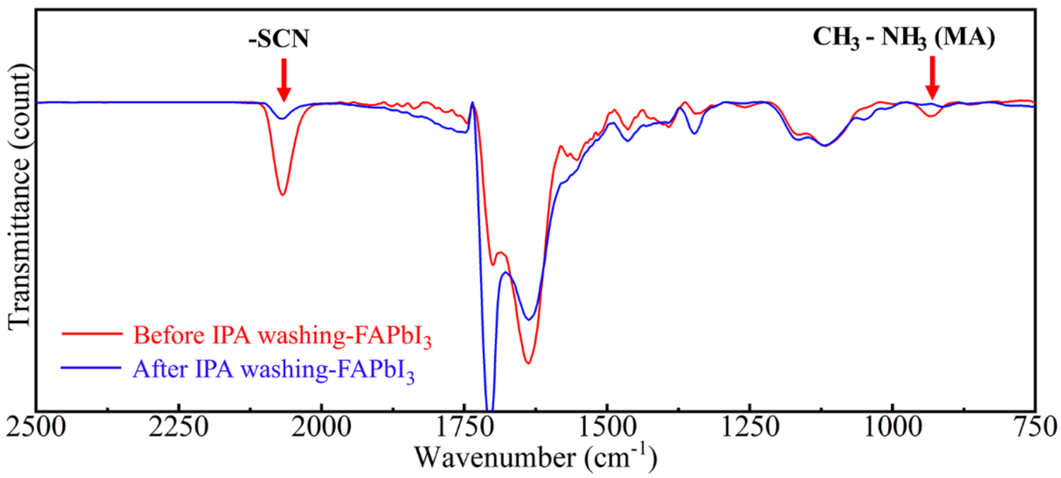

Figure S8. The FTIR spectra of the MASCN-treated $FAPbI_3$ film before and after IPA washing.

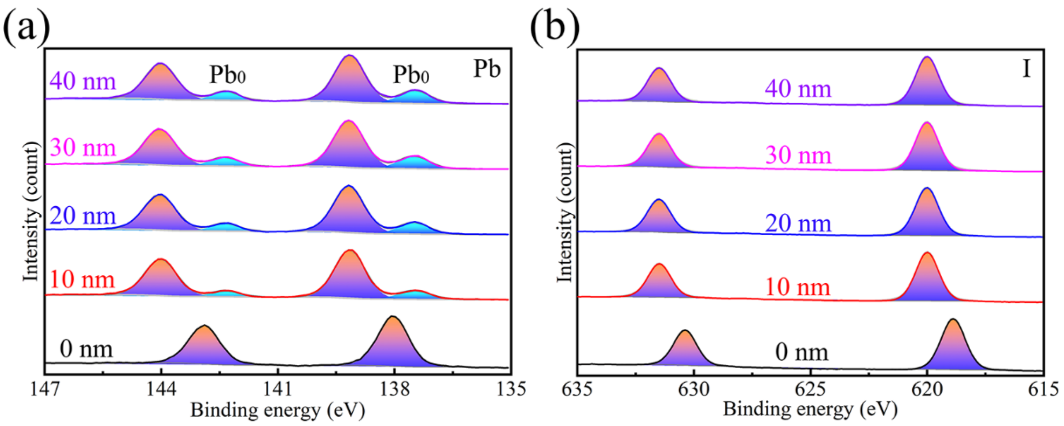

Figure S9. XPS pattern of a) Pb and b) I with different etching depths in a washed 0-min $FAPbI_3$ film.

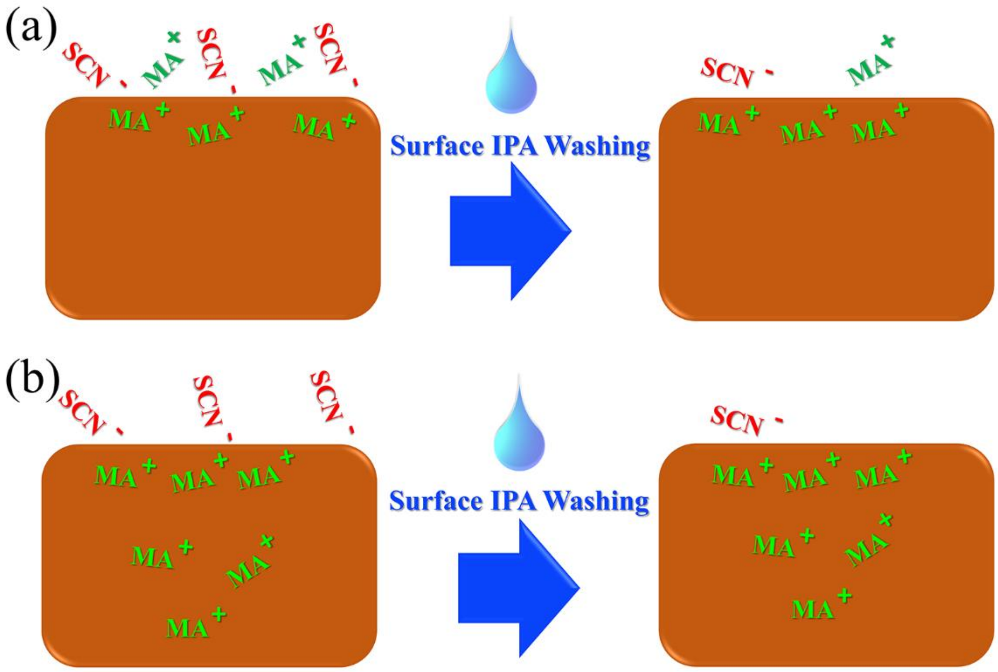

Figure S10. Effect of IPA washing on a) 0 min-$FAPbI_3$ and b) 10 min-$FAPbI_3$.

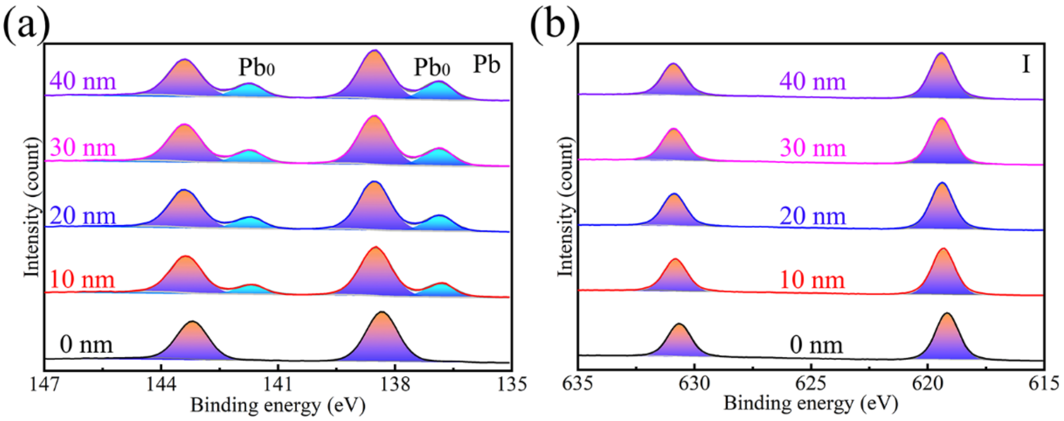

Figure S11. XPS spectra of a) Pb and b) I with different etching depths in a washed 10-min $FAPbI_3$ film.

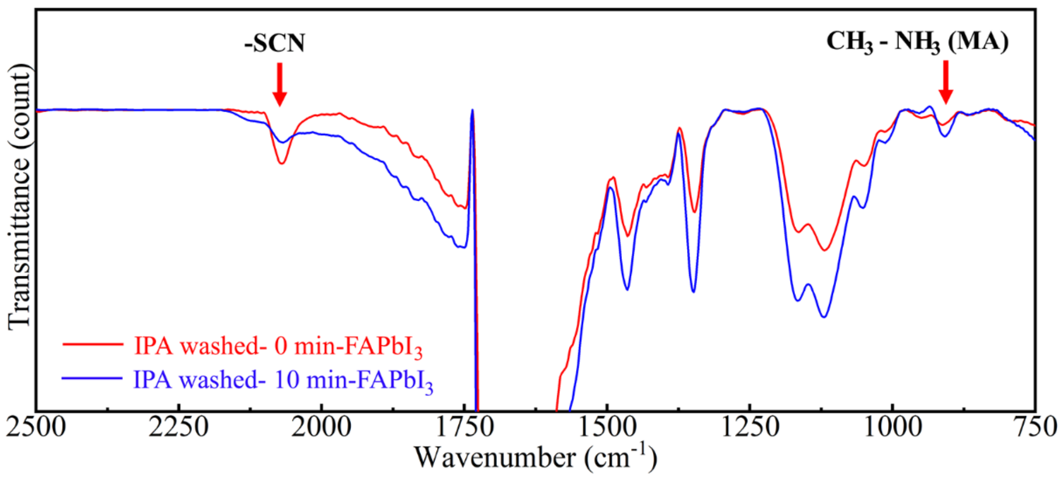

Figure S12. The FTIR spectra of the 0-min $FAPbI_3$ and 10 min $FAPbI_3$ films after IPA washing.

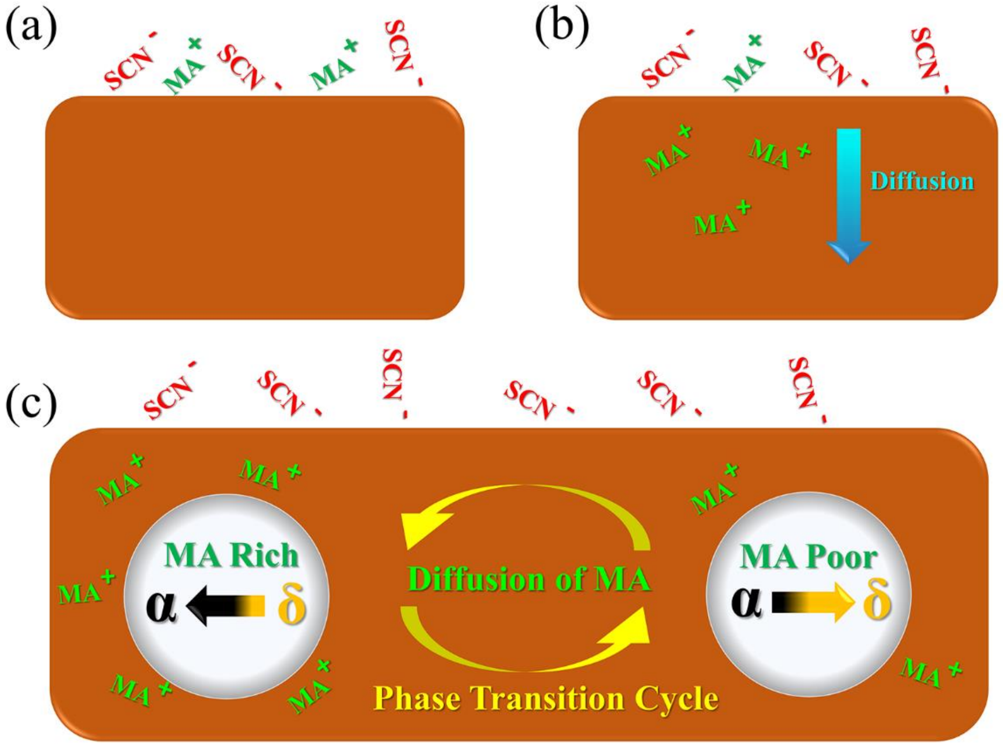

Figure S13. Schematic of the behavior of MA^+ and SCN^- ions. a) Initial state, b) diffusion process of MA^+, and c) resulting phase-transition-cycle process.

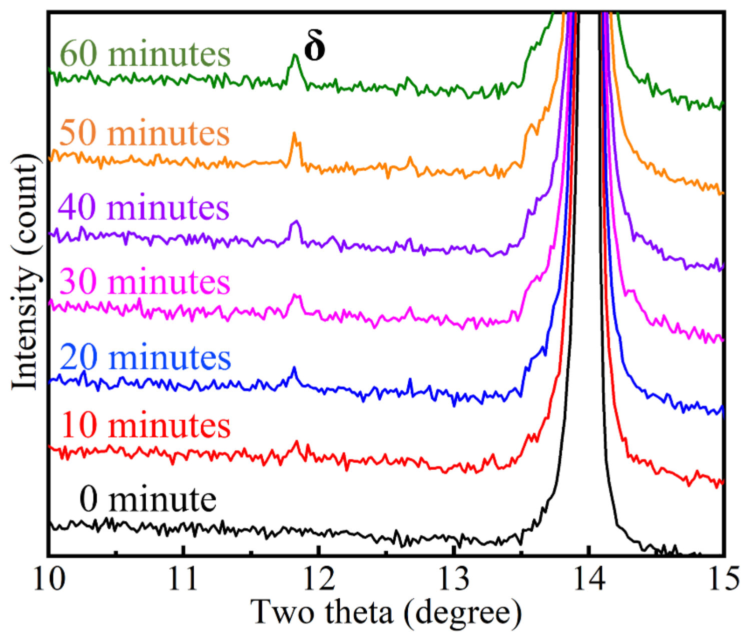

Figure S14. XRD pattern of the $FAPbI_3$ film treated with 10 mg mL^{-1} MASCN.

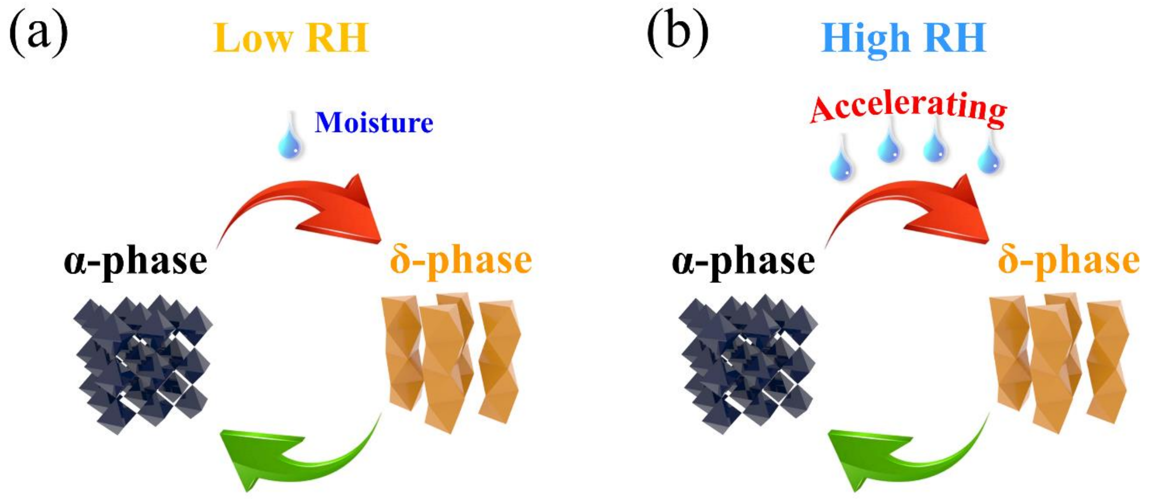

Figure S15. The effects of a) low and b) high RH on the PTC process.

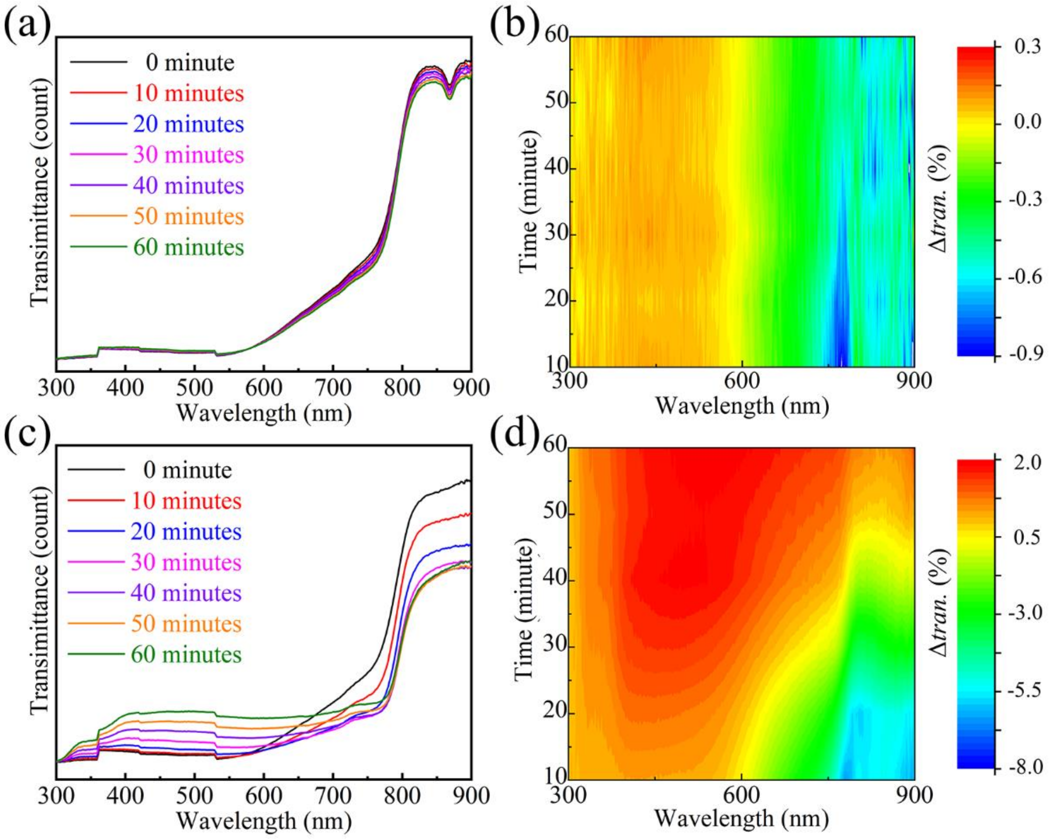

Figure S16. The transmittance and Δ*tran*. of the MASCN-treated $FAPbI_3$ film a,b) sealing in CB and c,d) under open air environment.

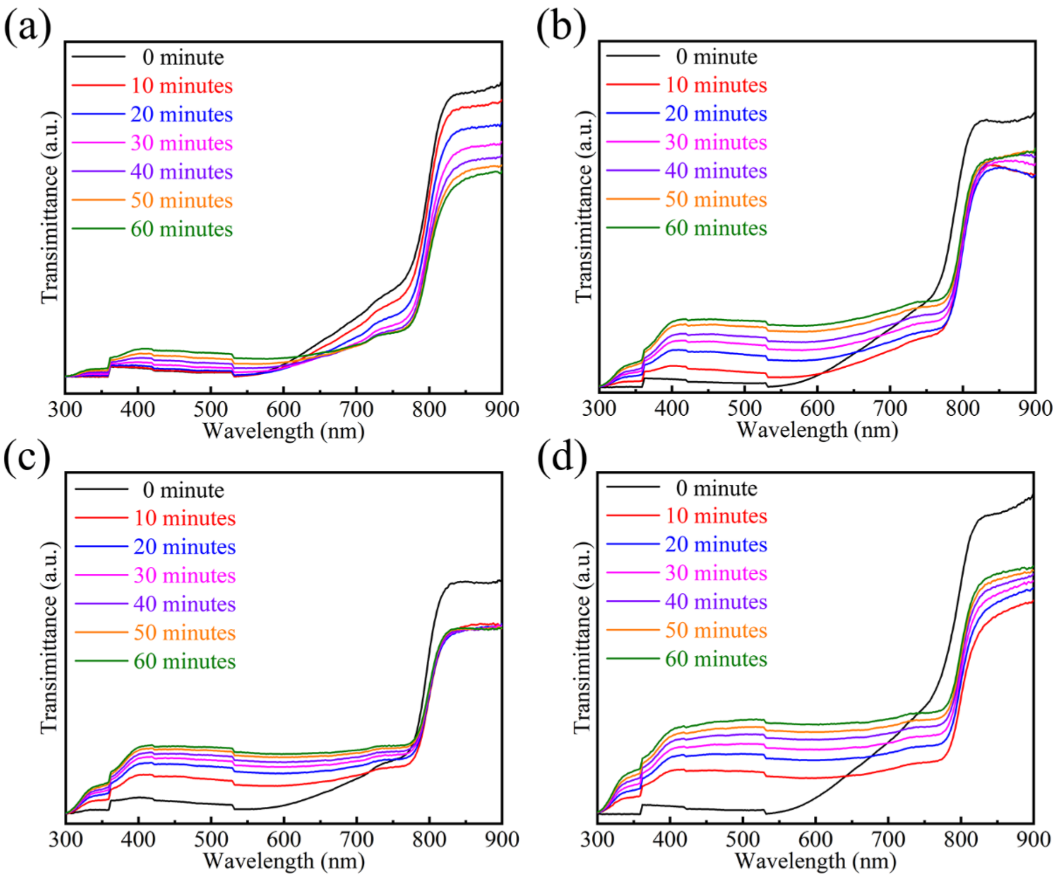

Figure S17. The transmittance variation of the MASCN-treated $FAPbI_3$ film under different RH of a) 0~5%, b) 20~25%, c) 50~55% and d) 80~85%.

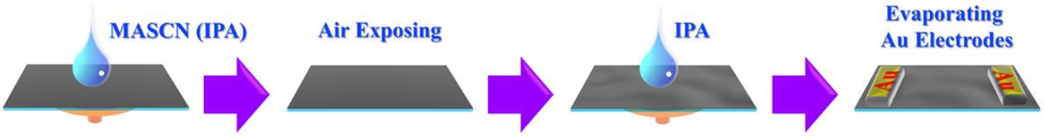

Figure S18. Schematic of the fabrication process.

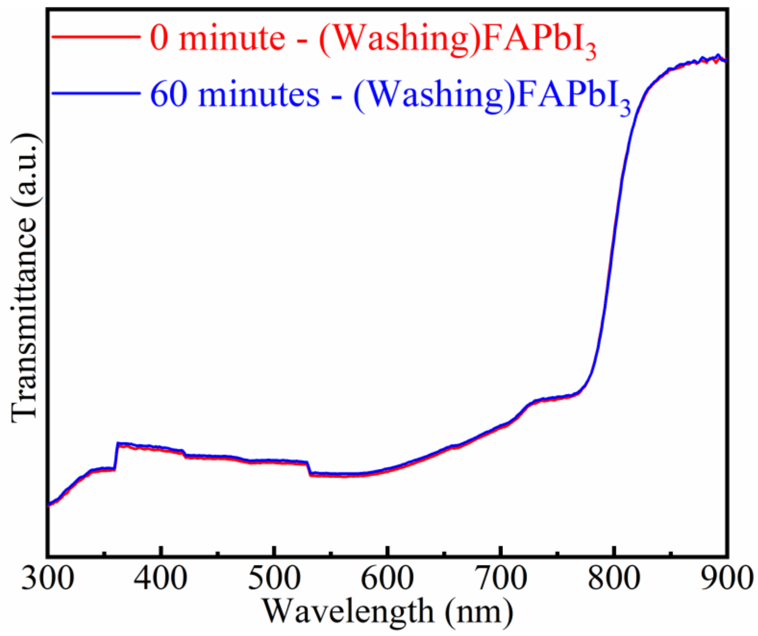

Figure S19. The transmittance spectra of the IPA-washed film after 0 and 60 minutes.

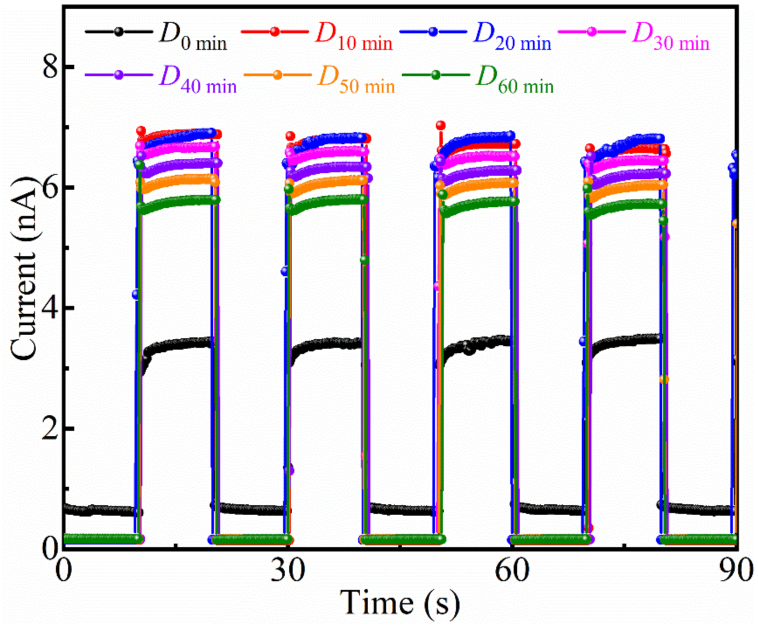

Figure S20. The *I*-*t* curves of the photodetector based on the *x*-min $FAPbI_3$, where *x* is from 0 to 60, respectively. The applied voltage is 1 V, and the wavelength of incident light is 650 nm.

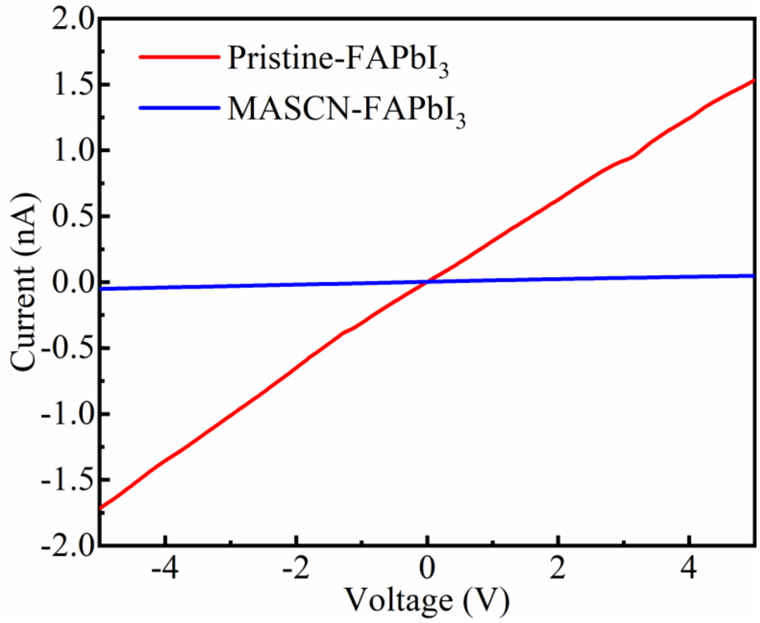

Figure S21. The *I-V* curve in the dark of the device based on the Pristine- and MASCN-$FAPbI_3$ films.

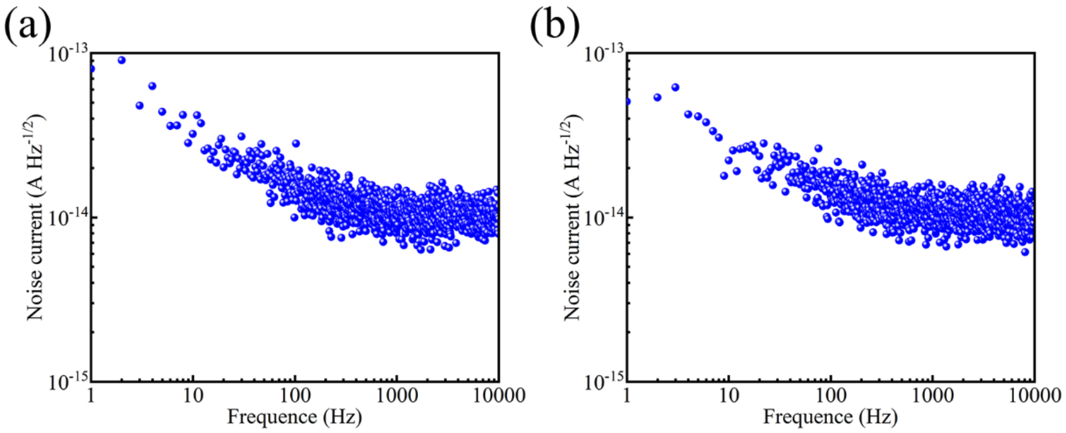

Figure S22. Frequency dependent noise current of the devices based on a) Pristine- and b) MASCN- $FAPbI_3$ measured at 1 V.

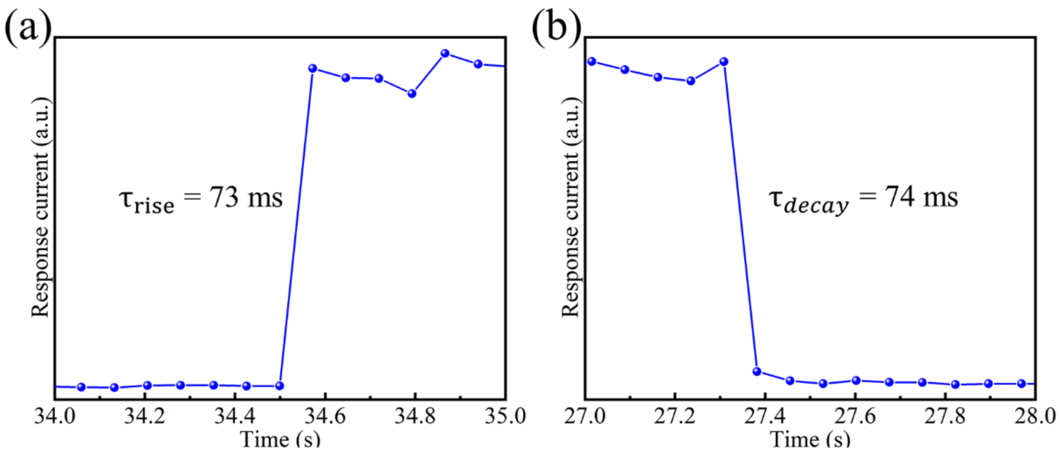

Figure S23. a) Rise and b) decay time of the device based on MASCN-$FAPbI_3$.

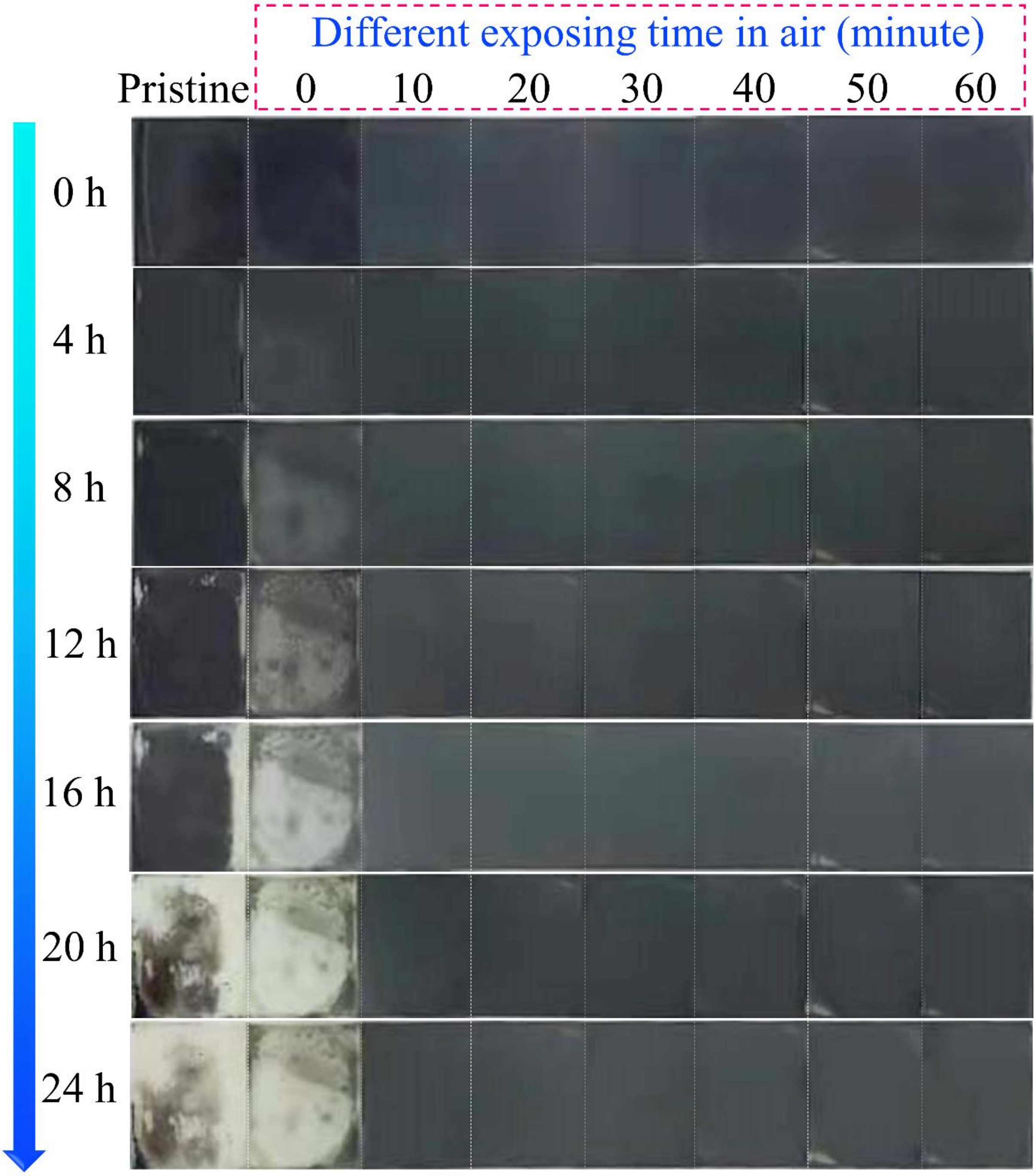

Figure S24. The stability of the Pristine-$FAPbI_3$ and IPA washed *x*-min $FAPbI_3$ film under a high RH over 90%.

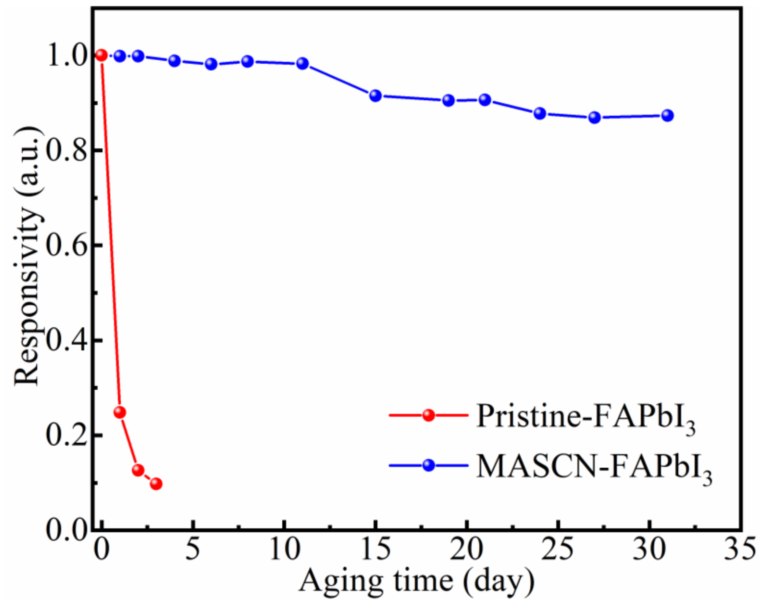

Figure S25. The stability of devices based on Pristine-$FAPbI_3$ and MASCN-$FAPbI_3$ film. The RH is controlled within 50% to 55%.

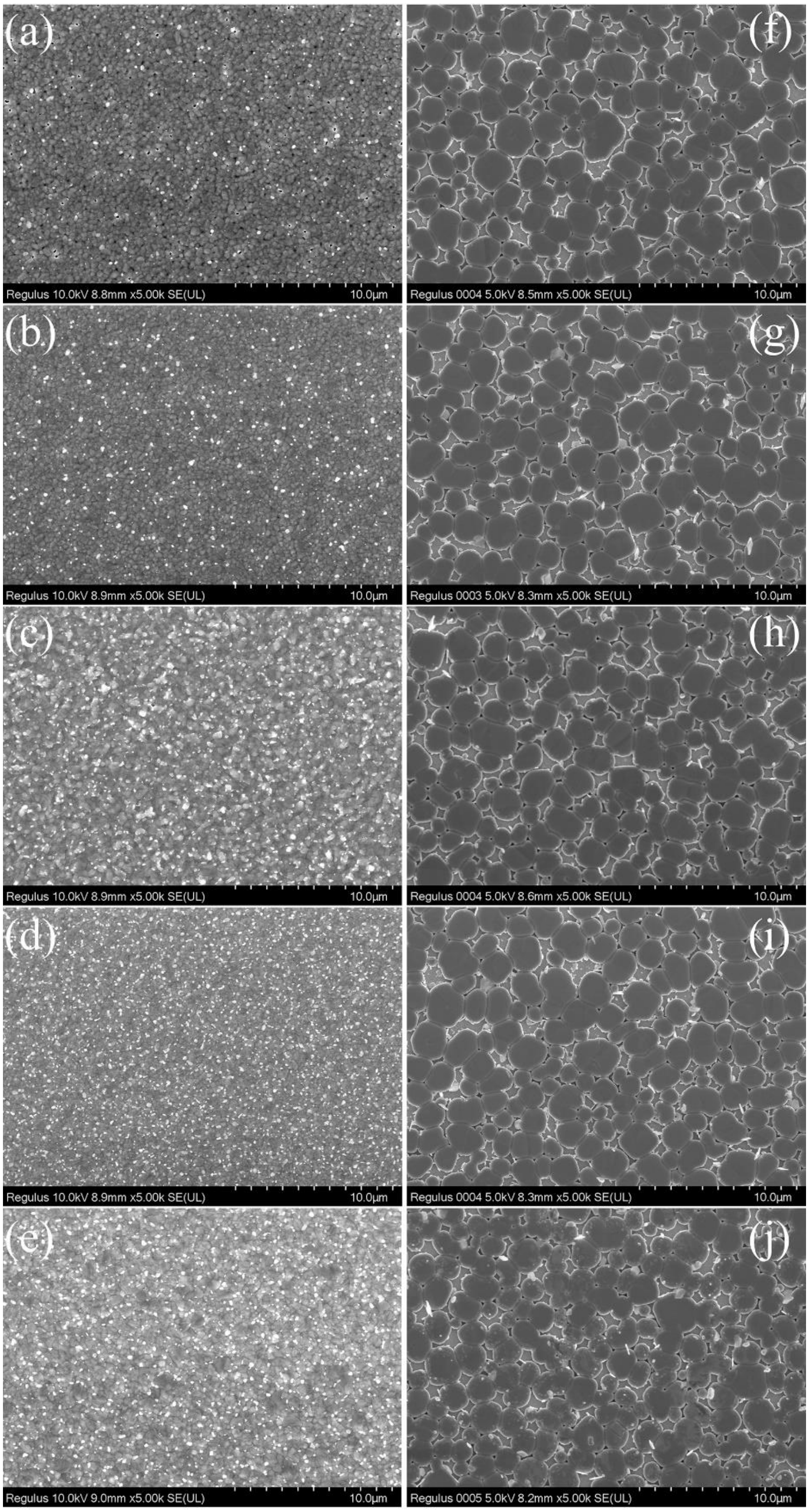

Figure S26. a-e) SEM images of the film fabricated with different antisolvent parameters from (14 s)- to (22 s)-$FAPbI_3$. f-j) SEM images of the MASCN-treated film corresponding to the film in the left line of a-e).

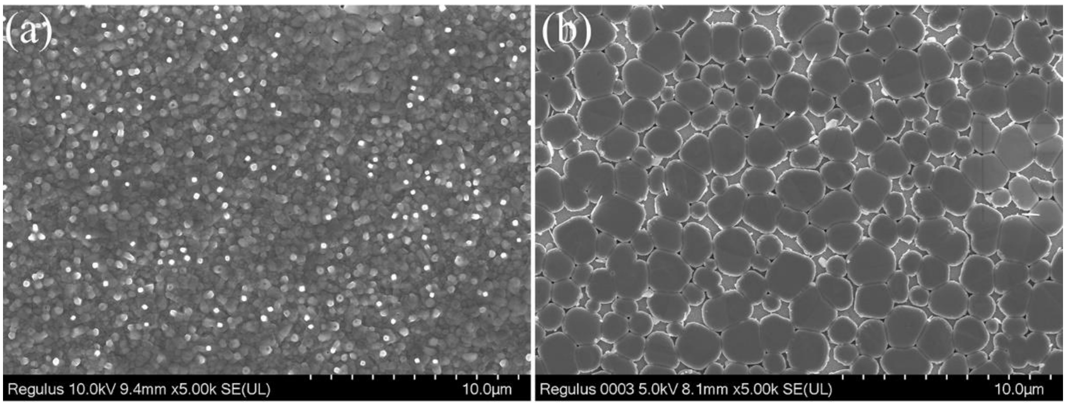

Figure S27. SEM images of the a) initial and b) MASCN-modified $FAPbI_3$ films fabricated by the two-step method.

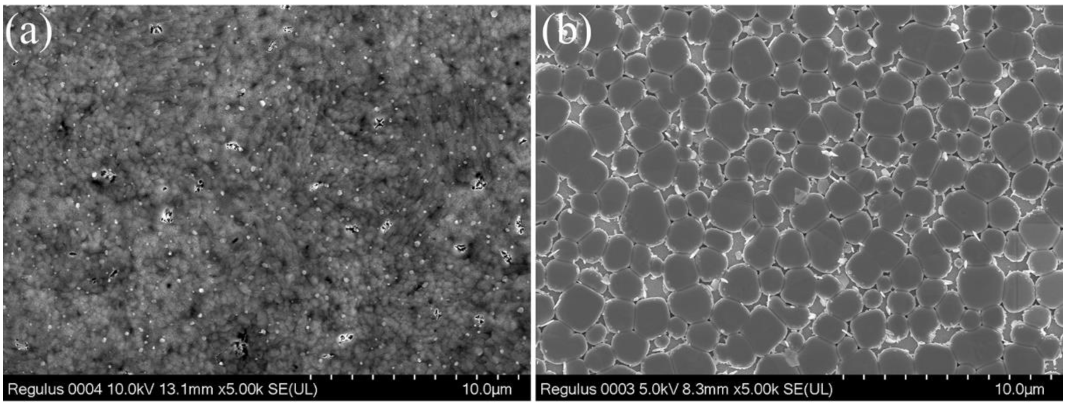

Figure S28. SEM image of the a) decomposed and b) saved $FAPbI_3$ films.

Supporting Table 1. The absolute value of Δ*tran.* at the wavelength of 500 nm with different exposure time under different RHs.

Δ*tran.* (%) / RH (%) / Time (minute)	0~5	20~25	50~55	80~85
0	0	0	0	0
10	0.124	0.535	10.739	13.516
20	0.184	5.25	2.593	3.865
30	0.732	2.244	1.826	1.515
40	0.988	1.592	1.341	1.351
50	1.401	1.55	1.099	1.128
60	1.72	1.621	0.794	1.632

Supplementary Note 1: The PL spectra of the MASCN-treated perovskite film

In Figure 1d, the time-dependent PL spectra of the MASCN-treated film under an open environment is shown. The PL intensity obviously increases with time, and there is also a tiny redshift of the intensity peaks. In Figure 2, we have proven that it is the MA group that dopes into the $FAPbI_3$ crystal, and normally, the dopes of MA into $FAPbI_3$ crystal will cause a blueshift because the bandgap will increase. Here, such a shift is attributed to the quantum size effect rather than bandgap variation.[1] Specifically, when the crystal size merges from a small crystal into a large crystal (Figure 1b), the bandgap of perovskite tends to become small, thus causing a redshift in the PL spectra. In this situation, the variation in the crystal size dominates the peak shift rather than MA doping. This can also be confirmed by the front and back PL spectra, where the shift occurs simultaneously at the top and bottom of the film (Figure S29). The MA group diffuses from the surface first, which is not consistent with the PL spectra.

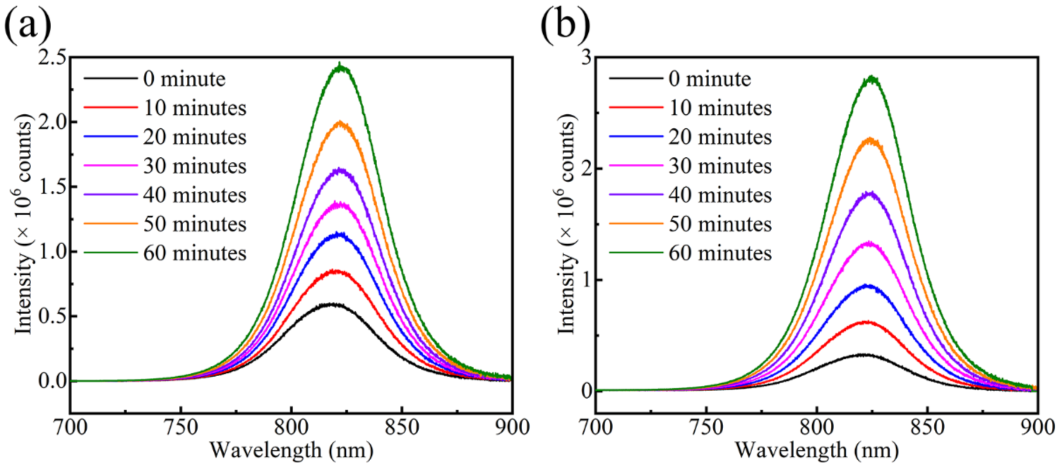

Figure S29. The time-dependent PL variation of the MASCN-treated $FAPbI_3$ film characterized from the a) front and b) back sides.

Supplementary Note 2: The different phase transition behaviors caused by FASCN and MASCN

In our work, FASCN causes a phase transition from α- to δ-$FAPbI_3$, while MASCN causes the reverse phenomenon. Considering that the FA^+ group has a tiny impact on the $FAPbI_3$ film, it is concluded that the SCN^- group will cause the phase transition from α- to δ-$FAPbI_3$, and the MA^+ group reverses this impact. Grätzel et al. provided a molecular dynamic simulation to explain the impact of MASCN on the surface of $FAPbI_{3,}$ and the results showed that surface interactions can also affect the crystal system energy.[2] Here, we find that the surface SCN^- treatment will change the lattice strain in the $FAPbI_3$ film, where the strains in the out-of-plane direction are increased (Figure S30).

In detail, the α-$FAPbI_3$ film is treated with different concentrations of FASCN, and all of them turn into δ-$FAPbI_3$. Interestingly, a redshift in the signals of the resulting δ-$FAPbI_3$ is observed, and the shift is increased with higher FASCN concentrations. According to the Bragg diffraction formula:

$$2\, d \sin \theta = n\, \lambda,$$

where d represents the interplanar spacing, θ represents the diffraction angle, n represents the diffraction series, and λ represents the wavelength of the X-ray. Thus, an increase in θ means a reduction in d, which means that the lattice plane in the out-of-plane is compressed by the interaction between FASCN and the perovskite crystal.

The space structure of the crystal phase is the result of the energy distribution, where the system energy is supposed to be minimized so that the structure can be stable. The strain

introduced by the SCN$^-$ group may change the balance in the $FAPbI_3$ phase and reduce the energy barrier in the phase transition, both α- to δ-type and δ- to α-type. When there is only an SCN group (FASCN), the α- to δ- transition occurs. However, when the MA group is involved, it has been widely reported that the doping of MA makes the α-phase more stable at room temperature;[3] thus, the δ- to α-transition occurs (MASCN).

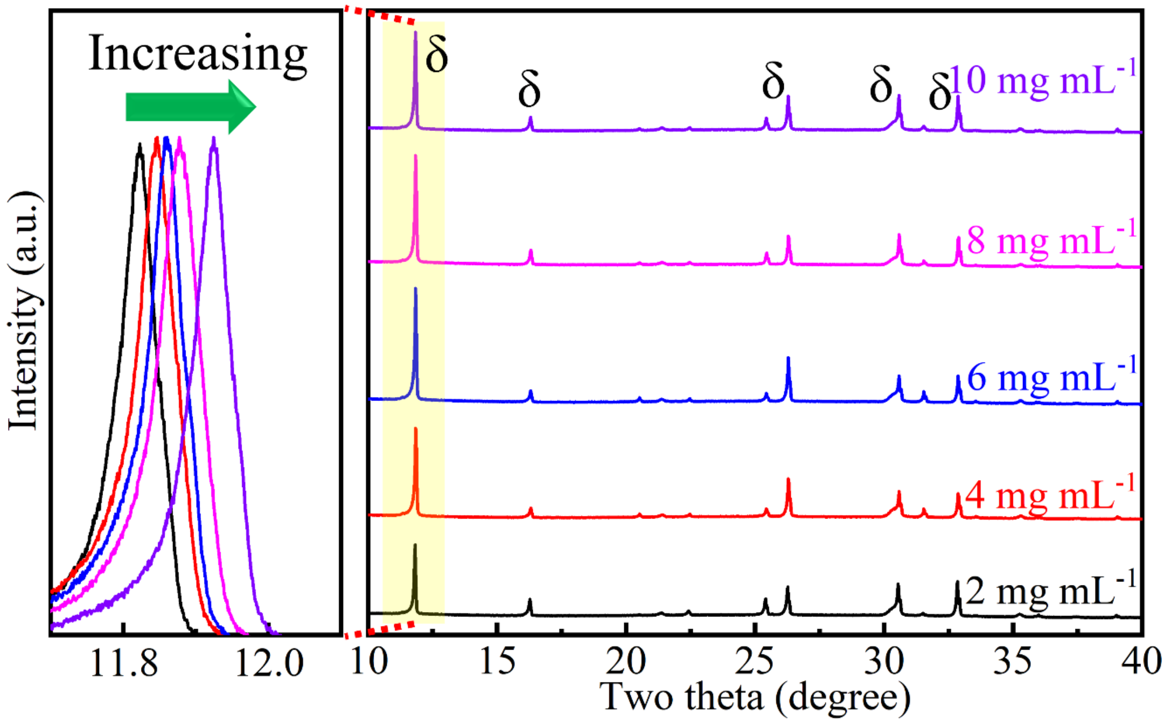

Figure S30. The XRD pattern of the resulting $FAPbI_3$ film fabricated with different FASCN treatment concentrations. The left graph shows the magnification of the diffraction angle from 11.7° to 12.1°.

Supplementary Note 3: The relationship between Δ*tran*. and spectra wavelength

During the MASCN-induced recrystallization process, there is competition between the two inducements in the variation of transmittance, which can be observed in Figure 4 and Figure S16. The first inducement is that when the film becomes mesoporous, its ability to reflect incident light will decrease, and transmittance will increase for the whole spectra. The second inducement is the change in the thickness of the film. For the initial state, the perovskite is a compact film with a thickness of approximately 450 nm. However, the recrystallization process will cause small crystals to merge into large crystals, with an increase in the thickness of the film. Cross-sectional SEM images show that the film after the recrystallization process can be up to several micrometers (Figure S31). The increase in thickness will strengthen the film absorption at the region around its absorption edge.[4] As a result, Δ*tran*. exhibits an obvious boundary in Figure S16b. However, when the RH is high, the effect of the first inducement will dominate Δ*tran*., and the absorption strength will terminate very quickly.

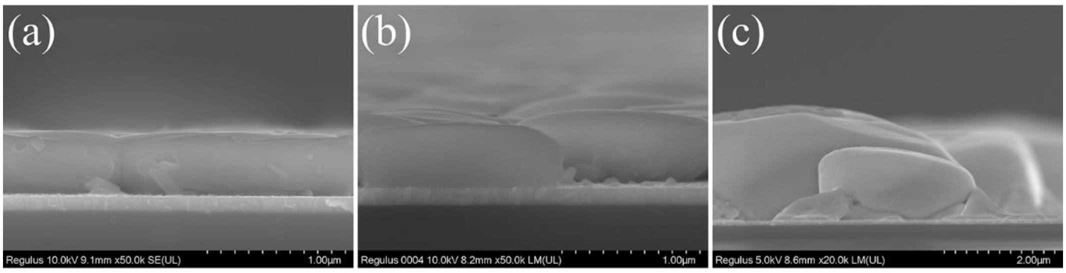

Figure S31. The cross-sectional SEM images of a) pristine $FAPbI_3$ film and after the recrystallization process for b) 10 minutes and c) 1 day.

Supplementary Note 4: The optoelectronic performance of $D_{x\text{-min}}$.

In Figure S20, the response current (I_{res}) of different $D_{x\text{-min}}$ is compared, and $D_{10\text{-min}}$ has the best performance. From $D_{0\text{-min}}$ to $D_{10\text{-min}}$, there is a large improvement in I_{res}. Modification of the perovskite crystal contributes to such improvement, which can be observed in Figure 5g. After this, from $D_{10\text{-min}}$ to $D_{60\text{-min}}$, the I_{res} gradually decreases with a small magnitude. Considering that the perovskite crystal is still optimized by the recrystallization process, this variation is caused by the gradually decreasing perovskite covering area, which can be observed in the absorption or transmittance spectra characterization and Figure 1.

Furthermore, the dark current of $D_{\text{x-min}}$ performs an obvious decrement from $D_{0\text{-min}}$ to $D_{10\text{-min}}$, and then also gradually decreases with a tiny magnitude (Figure S32). Such changes are attributed to two parts: First, crystallization is kept modified with time passing by (Figure 1e). Second, the shrinking perovskite film results in poor connection, which will decrease conductivity of the device. For lateral device, decreasing conductivity will reduce both response and dark current. Though low dark current will benefit properties such as detectivity, the decrement in Figure S20 is more significant due to the serious light absorption loss. Thus, $D_{10\text{-min}}$ is selected as the champion device.

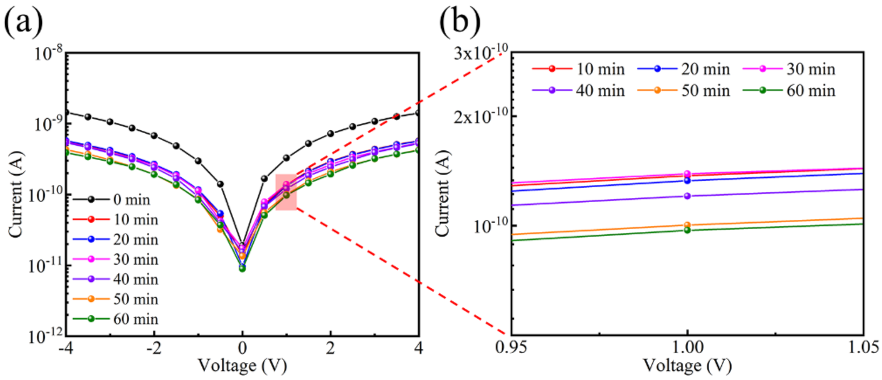

Figure 32. a) *I*-*V* curve of the devices with different exposure time under dark. b) the magnified figure of red area in a).

Supplementary Note 5: The temperature-dependent SCLC characterization

The temperature-dependent SCLC characterization is an effective method to extract the intrinsic distribution of the energy state in semiconductors.[5-8] The basic theory is Ohm's law and the Poisson equation, and the relationship between the energy state (N_s) and Fermi level (E_F) has been deduced in previous works as follows:

$$\frac{dN_S}{dE_F} = \frac{1}{k_BT}\frac{\varepsilon\varepsilon_0}{eL^2}\frac{(2m-1)}{m}(1 + C), (1)$$

where k_B is the Boltzmann constant, T is the temperature, ε is the dielectric constant of $FAPbI_3$, ε_0 is the permittivity of vacuum, e is the elementary charge, and L is the length of the film. $m = \frac{\mathrm{d\,In}\,J}{\mathrm{d\,In}\,V}$ is the logarithmic curve of J-V. and C is depicted as:

$$C = \frac{B(2m-1)+B(3m-2)+d\,[\mathrm{In}(1+B)]/\mathrm{d\,In}U}{1+B(m-1)}, (2)$$

where B contains higher ordered derivatives of J-V as follows:

$$B = -\frac{[\mathrm{d\ m/d\ In\ U}]}{m(m-1)(2m-1)}. (3)$$

Normally, Equation (1) can be calculated by measuring the *J*-*V* curve at a certain temperature, and C and B can be derived from the curve. However, it is also necessary to build the relationship between V and the energy level. This can be derived from the Arrhenius plot of the SCLC curves at a given V. In detail,

$$E_{\mathrm{A}} = \frac{\mathrm{d}\ \mathrm{In}\ J}{\mathrm{d}\ (\mathrm{k}_B T)^{-1}}, (4)$$

where T is the setting temperature, and J is the characterized at each given V. Equation (2) can be deconvolved with Equation (4) to extract the density of state (DOS).

[1] B. Li, Y. Zhang, L. Fu, T. Yu, S. Zhou, L. Zhang, L. Yin, *Nat. Commun.* **2018**, *9*, 1076.

[2] H. Lu, Y. Liu, P. Ahlawat, A. Mishra, W. R. Tress, F. T. Eickemeyer, Y. Yang, F. Fu, Z. Wang, C. E. Avalos, B. I. Carlsen, A. Agarwalla, X. Zhang, X. Li, Y. Zhan, S. M. Zakeeruddin, L. Emsley, U. Rothlisberger, L. Zheng, A. Hagfeldt, M. Grätzel, *Science* **2020**, *370*, 8985.

[3] M. Kim, G.-H. Kim, T. K. Lee, I. W. Choi, H. W. Choi, Y. Jo, Y. J. Yoon, J. W. Kim, J. Lee, D. Huh, H. Lee, S. K. Kwak, J. Y. Kim, D. S. Kim, *Joule* **2019**, *3*, 2179.

[4] J. Wang, S. Xiao, W. Qian, K. Zhang, J. Yu, X. Xu, G. Wang, S. Zheng, S. Yang, *Adv. Mater.* **2021**, *33*, 2005557.

[5] V. Adinolfi, M. Yuan, R. Comin, E. S. Thibau, D. Shi, M. I. Saidaminov, P. Kanjanaboos, D. Kopilovic, S. Hoogland, Z. H. Lu, O. M. Bakr, E. H. Sargent, *Adv. Mater.* **2016**, *28*, 3406.

[6] C. Krellner, S. Haas, C. Goldmann, K. P. Pernstich, D. J. Gundlach, B. Batlogg, *Phys. Rev. B* **2007**, *75*, 245115.

[7] J. Dacuña, A. Salleo, *Phys. Rev. B* **2011**, *84*, 195209.

[8] F. Schauer, R. Novotny, S. Nešpůrek, *J. Appl. Phys.* **1997**, *81*, 1244.
